# Association of pre-migration socioeconomic status and post-migration mental health in Syrian refugees in Lebanon: a descriptive sex-stratified cross-sectional analysis

**DOI:** 10.1186/s41256-024-00347-0

**Published:** 2024-03-04

**Authors:** Saskia Lange, Toivo Glatz, Andreas Halgreen Eiset

**Affiliations:** 1 Institute of Public Health, Charité – Universitätsmedizin Berlin, Corporate Member of Freie Universität Berlin and Humboldt-Universität zu Berlin, Charitéplatz 1, 10117 Berlin, Germany; 2 Department of Public Health, Center for Global Health (GloHAU), Aarhus University, Aarhus, Denmark; 3 Clinic for PTSD and Anxiety, Aarhus University Hospital, Aarhus, Denmark; 4 Department of Clinical Pharmacology, Aarhus University Hospital, Aarhus, Denmark

**Keywords:** Anxiety, Depression, Mental health, Migration, Refugees, SES, Sex, Gender, Social determinants

## Abstract

**Background:**

Refugee populations present with high levels of psychological distress, which may vary among sociodemographic characteristics. Understanding the distribution across these characteristics is crucial to subsequently provide more tailored support to the most affected according to their specific healthcare needs. This study therefore seeks to investigate the association between pre-migration socioeconomic status (SES) and post-migration mental health separately for male and female Syrian refugees in Lebanon.

**Methods:**

In a cross-sectional study, a cluster randomized sample of 599 refugees from Syria were recruited between 2016 and 2019 within 12 months after they fled to Lebanon. Logistic regression was used to determine the association between self-reported pre-migration SES and levels of anxiety and depressive symptoms assessed on the Hopkins Symptoms Checklist-25 (HSCL-25) scale, both for the entire sample and stratified by sex. To assess the informative value of self-reported SES, its correlation with education variables was tested. All analyses were conducted in R version 4.3.

**Results:**

Using complete cases, 457 participants (322 female, 135 male) were included in the analyses. Females showed on average more symptoms of anxiety (Median: 2.5) and depression (Median: 2.4) than males (Median: 2.10 and 2.07, respectively). Below average SES was associated with significantly higher odds for mental illness compared to average SES (anxiety: OR 4.28, 95% CI [2.16, 9.49]; depression: OR 1.85, 95% CI [1.06, 3.36]). For anxiety, differences between SES strata were larger for males than females. The self-reported SES measure showed only a weak positive correlation with education.

**Conclusions:**

This study adds additional descriptive data highlighting mental health differences in Syrian refugees in Lebanon, whereby below average SES is associated with worse mental health outcomes compared to average SES. These findings demand further research into the underlying mechanisms. Improving our understanding of the observed differences will provide valuable insights that can contribute to the future development of targeted measures.

**Supplementary Information:**

The online version contains supplementary material available at 10.1186/s41256-024-00347-0.

## Background

The number of forcibly displaced persons (FDPs) worldwide climbed to a record high in May 2022 with more than 100 million people having been forced to flee armed conflict, violence, human rights violations, and persecution [1]. Refugees and asylum seekers constitute more than one third of FDPs. Potentially traumatic events, like experiences of war and conflict which precipitated fleeing, in and of themselves already pose a risk to the mental health of FDPs [2]. Circumstances like separation from family, difficult and potentially dangerous migration routes and challenges with resettlement further affect mental health negatively [3, 4]. In addition, post-migration stressors like poor social integration and support, discrimination, lack of language skills, and financial difficulties contribute to the development of mental ill health [3, 5, 6]. These factors play a particularly important role shortly after migration and their impact can—under favorable conditions—decrease over time so that incidence rates of mental illness among refugees have often, yet not always, been shown to decrease over time after migration [7]. However, the actual development ultimately depends on a wide variety of social determinants—individual and contextual—which can have both a positive and a negative influence on the overall trend [3, 7]. Mental health outcomes among refugees and asylum seekers may thereby also vary depending on whether they are resettled in high-income or low-income countries, as systematic reviews have shown that prevalence estimates of mental health disorders differ strongly across all countries of origin and diverse host countries [8], but studies on this specific relation are rare.

Nevertheless, the prevalence of mental illness among refugees and asylum seekers in general is consistently high and a recent meta-analysis estimated the pooled prevalence of posttraumatic stress disorder (PTSD) at 31%, depression at 32%, and anxiety at 11% across all countries of origin and diverse host countries [4]. Individual studies focusing on the mental health of adult Syrian refugees, who comprise the largest group of refugees worldwide found large differences in estimates for PTSD, depression and anxiety across different host countries [6, 9, 10], with PTSD prevalence estimates ranging from 11.4% in Germany [9] to 55% in Lebanon and 60% in Denmark [10]. These studies assessed outcomes mostly between 2 and 5 years after migration.

To develop targeted measures that reach those with the greatest needs, refugees cannot be considered a homogenous group. The considerable differences in mental health outcomes among Syrian refugees in various host countries mentioned above might be the result of not only varying migration experiences and structures in the host countries, but also factors such as social class, sex and gender that are now widely acknowledged to influence (mental) health [11, 12]. Social class and sex/gender being key dimensions, we will elaborate on their specificities and their potential implications for mental health outcomes in the following sections.

Socioeconomic status (SES) is often the preferred instrument to measure social class and it has been operationalised as a derived measure of education, income and occupation or as a compound of several [13] which can be combined in a composite score, but also analyzed individually [14]. Besides measuring SES using these dimensions, there is also the option of assessing it using respondents' self-evaluation. A recent meta-analysis found that self-assessed SES had moderate agreement with measured SES and it has been suggested that self-assessed SES can be used to specifically capture the aspect of perceived social position [15]. In general, SES is thought to be positively associated with mental health [16, 17] but the direction of causality and its mechanisms, as well as the generalizability of this association across populations, is not completely clear and understood [13].

The terms sex and gender are often used interchangeably in public health research, while in fact they denote two very different concepts. Sex is determined by biological factors like chromosomes, hormones and genitalia, whereas gender refers to social aspects like gender roles or gender expression [18]]. A conflation of these terms and methodological inconsistencies hinders our understanding of health differences among sexes and genders [19, 20], thus preventing effective action to counter these. Finally, both sex and gender are often binarily assessed, excluding those who do not fit into this binary [21, 22]. Yet, in the rare cases where non-binary and intersex individuals are explicitly considered, they present with the greatest mental health burden of all sexes/genders, as has been shown for mental health status during the Covid-19 pandemic [23].

Only few studies exist on the relation between SES or any of its subdimensions and mental health among refugees which consider differences by sex or gender. In the following presentation of two studies’ results, the original terms (sex/gender) that were used in these studies will be deployed respectively. It should be kept in mind, however, that the use of the terms seems rather unspecific and arbitrary in both cases. A study by Bauer and colleagues found that the overall level of health satisfaction after migration, which encompasses mental health, was lower for women than for men, despite comparable levels before migration. However, the study does not report information on the statistical significance of differences in health satisfaction by pre-migration SES and sex combined. Another cross-sectional, population-based study examined the mental health of refugees and non-refugee migrants from various countries in Sweden [25]. Individuals with more years of education were less likely to purchase psychotropic drugs (used by the authors as a proxy for mental problems) than those with fewer years of education. In a gender-stratified analysis it could furthermore be seen that this pattern was more pronounced in women than in men. However, this study did not differentiate between refugees and non-refugee migrants for all of their analyses, and years of education represent only one part of the SES [25]. This relationship might therefore not be reproduced when taking another dimension of the SES or the complete construct.

In order to provide better support through health care and policies for refugees, we need to better understand the distribution of mental health problems and refugees’ corresponding health care needs. Therefore, this study aims to investigate the relation of self-reported pre-migration SES and symptoms of anxiety and depression shortly after migration in a study sample of Syrian refugees in Lebanon for males and females separately as an example for migration from and to a low and middle income country (LMIC). More specifically, the aim is to answer the following question:

Is pre-migration SES associated with post-migration mental health in male and female Syrian refugees in Lebanon?

### Hypothesis 1

Higher pre-migration SES is associated with better post-migration mental health, i.e., less symptoms of anxiety and depression, in Syrian refugees in Lebanon.

### Hypothesis 2

The strength of this association is different for males and females.

These hypotheses are based on previous findings that higher SES is positively associated with better mental health, both in the general and in refugee and migrant populations, as well as observed sex differences as outlined above [16, 24]. The available evidence is currently limited and inconclusive, however, which is why any formulated hypotheses should be considered exploratory rather than confirmatory.

## Methods

### Study design and setting

This work presents a secondary analysis of primary data that were collected in the cross-sectional “Asylum seekers’ and refugees’ changing health” study between 2016 and 2019 in Lebanon [26].

In partnership with local non-governmental organizations, Lebanon was delineated into five regions utilizing a multifaceted stratification approach taking into account geographical factors, infrastructural elements, and insights about local complexities, such as political and religious dynamics. The sampling frame was crafted to encompass formal and informal refugee camps, as well as urban and rural communities, settlements, and congregations of Syrian refugees. Site selection for inclusion followed a randomized methodology aimed at proportional allocation within each stratum, constituting a stratified cluster sampling design.

### Study population

Participants had to be at least 18 years old and have arrived at the host country within 12 months prior to study participation. After the randomized site selection, all individuals deemed eligible for participation, primarily facilitated through oral communication, were invited to partake in the study, thereby ensuring proportional allocation of participants across the selected sites. Ultimately, 599 participants residing in refugee camps in Lebanon were included in the study.

### Variables and measurement

For all participants, sociodemographic information, migration history, and mental health were assessed by questionnaires and standardized scales. The language of examination was Syrian Arabic. The following variables relevant to this study’s purpose were collected:

The dependent variables in this study are symptoms of anxiety and depression after migration as measured by the Hopkins Symptom Checklist-25 (HSCL-25) [27]. The HSCL-25 is a screening tool validated for different languages and cultural contexts including Arabic [28, 29]. It uses 25 items (10 for anxiety symptoms, 15 for depressive symptoms) with response options on a 4-point Likert scale (from 1: “Not at all” to 4: “Extremely”). Each of the anxiety and depression scores were calculated by averaging the domain specific items. These scores were then dichotomized along the widely used and validated threshold of 1.75 points [29, 30], with values ≥ 1.75 points indicating levels of symptoms of anxiety or depression with a severity that would likely qualify for being diagnosed, i.e., being clinically relevant, if formally assessed by a trained health care professional. Thus, although our data were obtained using a screening tool, we will refer to values equal or above the threshold as “clinically relevant levels of symptoms” in line with the recommended interpretation of this tool’s results [30]. All analyses were run separately for anxiety and depression, using only the dichotomized values (i.e., presence vs. absence of clinically relevant levels of symptoms).

The independent variable of interest is SES. For analysis in this study, self-reported pre-migration SES, assessed retrospectively after migration, was used. Participants indicated whether their SES was below, on, or above average before their flight. There was an additional answer option “do not know/do not wish to answer”.

Sex is included as a binary variable. It was assessed by asking study participants to state their sex, with the two response options “male” or “female”.

### Statistical analyses

All analyses were conducted in R version 4.3 [31]. The R code as well as a comprehensive report of the statistical analysis are provided in the Additional files 1 and 2.

Descriptive statistics on the mentioned variables and additional sociodemographic characteristics were calculated for the total population as well as stratified by sex. Given the skewed distributions of the continuous variables, the median alongside minimum and maximum values were chosen to describe those variables. For ordinal and categorical variables, absolute numbers and proportions are presented for each category. Furthermore, the amount of missing data points was ascertained for each variable. The amount of missing data points is presented, and visualizations of missing data across variables were conducted to assess the missingness mechanism (see Additional file 3: Supplement 1).

To answer our research questions, we fitted eight logistic regression models: for each of symptoms of anxiety and symptoms of depression, four models were built giving the crude (one), the sex-stratified (two) and the crude including interaction-term of SES and sex (one) estimates. Interactions were interrogated by pairwise post-hoc comparisons by sex using least-square means from the *emmeans* package in R version 4.3 [32] adjusting *p* values for multiple comparisons using Bonferroni’s method for 4 comparisons. The reference levels used were SES “on average”, HSCL-25 scores below 1.75 points (absence of clinically relevant levels of symptoms), and female sex (only applicable in the latter two models). Missing observations in the SES variable were included in the “Do not know/do not wish to answer” category. No other treatment of missing variables was done in this analysis, thus approximating a “complete case” analysis.

To examine the informative value of the SES measure, the correlation of the self-reported SES with “years of education” (continuous) and “highest level of education” (ordinal, with levels from “no education” to “higher education”) was assessed by graphical exploratory techniques plotting these three variables pairwise against each other in separate figures, and by calculating Kendall’s Tau rank correlation coefficients. The answer category “Do not know/do not wish to answer” of the SES variable was excluded for these calculations.

To assess the results’ sensitivity to the handling of missing data, missing data for the HSCL-25 variables were forced into their extremes of absence of clinically relevant levels of symptoms and, in a second step, presence of clinically relevant levels of symptoms, and the same models as in the main analysis were run on these data.

To assess the results’ sensitivity to the selected threshold for the HSCL-25 variables, the analyses were re-run with a threshold of 2.0 for the HSCL-25 anxiety variable 2.1 for the HSCL-25 depression variable as suggested in a study conducted among an Arabic population of women in Beirut, Lebanon [33].

## Results

### Sample description

Descriptive data are presented in Table 1. The study population consists of 599 individuals in total, of which 408 are female and 153 male (38 missing), who have been in Lebanon for a median length of 8 months. The median age is 35 years.

**Table 1:** Baseline characteristics of the study sample stratified by sex

Variable	Total (n = 599)	Female (n = 408)	Male (n = 153)	Sex missing (n = 38)
Age				
Median [min, max]	35 [18, 90]	34 [18, 71]	38 [18, 90]	35 [25, 53]
Missing: n (%)	58 (9.7%)	27 (6.6%)	2 (1.3%)	29 (76.3%)
SES				
n (%)				
Below average	137 (22.9%)	90 (22.1%)	43 (28.1%)	4 (10.5%)
On average	341 (56.9%)	245 (60.0%)	90 (58.8%)	6 (15.8%)
Above average	24 (4.0%)	18 (4.4%)	6 (3.9%)	0 (0%)
Do not know/do not wish to answer	36 (6.0%)	30 (7.4%)	6 (3.9%)	0 (0%)
Missing	61 (10.2%)	25 (6.1%)	8 (5.2%)	28 (73.7%)
Education-years				
Median [min, max]	6 [0, 21]	6 [0, 20]	6 [0, 21]	9 [9]
Missing: n (%)	195 (32.6%)	121 (29.7%)	37 (24.2%)	37 (97.4%)
Education-highest degree				
n (%)				
No education	188 (31.4%)	131 (32.1%)	46 (30.1%)	11 (28.9%)
Primary school	246 (41.1%)	172 (42.2%)	73 (47.7%)	1 (2.6%)
High school	109 (18.2%)	82 (20.1%)	25 (16.3%)	2 (5.3%)
Higher	29 (4.8%)	20 (4.9%)	9 (5.9%)	0 (0%)
Missing	27 (4.5%)	3 (0.7%)	0 (0%)	24 (63.2%)
HSCL-25 anxiety score				
Median [min, max]	2.40 [1.00, 4.00]	2.50 [1.00, 4.00]	2.10 [1.00, 3.80]	2.20 [1.50, 2.80]
n (%)				
< 1.75	111 (18.5%)	60 (14.7%)	49 (32.0%)	2 (5.3%)
≥ 1.75	434 (72.5%)	323 (79.2%)	98 (64.1%)	13 (34.2%)
Missing	54 (9.0%)	25 (6.1%)	6 (3.9%)	23 (60.5%)
HSCL-25 depression score				
Median [min, max]	2.33 [1.00, 4.00]	2.40 [1.00, 4.00]	2.07 [1.00, 3.67]	2.07[1.47, 2.47]
n (%)				
< 1.75	99 (16.5%)	48 (11.8%)	50 (32.7%)	1 (2.6%)
≥ 1.75	380 (63.4%)	283 (69.4%)	85 (55.6%)	12 (31.6%)
Missing	120 (20.0%)	77 (18.9%)	18 (11.8%)	25 (65.8%)
Duration of stay in Lebanon (months)				
Median [min, max]	8.0 [1.0, 12.0]	8.0 [1.0, 12.0]	8.0 [4.0, 12.0]	8.0 [5.0, 12.0]

The proportional distribution of participants across SES levels is comparable for males and females, with the majority with an average SES (females: 60.0%, males: 58.8%), and least with an above average SES (females: 4.4%, males: 3.9%). Medians of the HSCL-25 sub-scores are higher among females than among males, both for anxiety (females: 2.50, males: 2.10) and depression (females: 2.40, males: 2.07).

### Missing data patterns

Most missing data can be observed for the variable “years of education” (32%), followed by the HSCL-25 depression score (20%), and then comparable levels for SES, age, and HSCL-25 anxiety score (9–10%). For SES, however, missing observations and those in the category “Do not know/do not wish to answer” taken together amount to 16%. Missing data is equally distributed across SES levels. Missingness of some variables seems to be related to each other, e.g., in 23 cases all relevant variables are missing, and in 21 cases years of education and information on the HSCL-25 depression are missing. A more detailed discussion of missingness mechanisms can be found in the Additional file 3, Supplement 1.

### Main analysis results

For the main analysis, 457 individuals have complete data (with missing SES data points being included in the “Do not know/do not wish to answer” category). Table 2 shows the number of participants per SES level and stratified by sex (for full baseline characteristics of included participants, see Additional file 3: Supplement 2).

**Table 2:** Participants included in the main analysis by SES level and sex

SES	Total (n = 457)	Female (n = 322)	Male (n = 135)
Below average	118 (25.8%)	78 (24.2%)	40 (29.6%)
On average	272 (59.9%)	194 (60.2%)	78 (57.8%)
Above average	18 (3.9%)	13 (4.0%)	5 (3.7%)
Do not know/do not wish to answer	49 (10.7%)	37 (11.5%)	12 (8.9%)

Results of the regression models are presented in Table 3 (anxiety) and Table 4 (depression). To test the first hypothesis, we consider the columns on the total population. In all six models, below average SES was associated with higher anxiety and depression scores compared to average SES (OR ranges: anxiety 2.64–10.57; depression 1.85–2.16). Above average SES was mostly associated with higher anxiety scores (OR ranges: anxiety 1.21–3.43; depression 0.71–1.16) but due to the small sample sizes in these categories, these estimates are very imprecise. The ORs for above average SES were generally smaller than those for below average SES. Missing information on SES was associated with higher anxiety and depression scores (OR ranges: anxiety 1.71–1.82; depression 1.75–2.32) (Table 3 and 4).

**Table 3:** ORs for HSCL-25 anxiety score ≥ 1.75 by SES, total and stratified by sex

SES	Total		Female		Male	
	OR [95% CI]	*p* value	OR [95% CI]	*p* value	OR [95% CI]	*p* value
Below average	4.28 [2.16, 9.49]	< 0.001	2.64 [1.14, 7.23]	0.036	10.57 [3.44, 46.37]	< 0.001
On average	1 (ref)	–	1 (ref)	–	1 (ref)	–
Above average	1.77 [0.56, 7.79]	0.380	1.21 [0.31, 8.05]	0.809	3.43 [0.48, 68.71]	0.280
Do not know/do not wish to answer	1.81 [0.85, 4.33]	0.148	1.82 [0.67, 6.38]	0.288	1.71 [0.50, 6.86]	0.409

Unadjusted ORs. Reference levels: HSCL-25 anxiety score < 1.75 points, average SES

**Table 4:** ORs for HSCL-25 depression score ≥ 1.75 by SES, total and stratified by sex

SES	Total		Female		Male	
	OR [95% CI]	*p* value	OR [95% CI]	*p* value	OR [95% CI]	*p* value
Below average	1.85 [1.06, 3.36]	0.035	2.16 [0.96, 5.51]	0.080	2.04 [0.91, 4.79]	0.091
On average	1 (ref)	–	1 (ref)	–	1 (ref)	–
Above average	0.87 [0.31, 2.78]	0.793	0.71 [0.20, 3.28]	0.615	1.16 [0.18, 9.17]	0.875
Do not know/do not wish to answer	2.00 [0.91, 5.05]	0.108	1.75 [0.64, 6.16]	0.318	2.32 [0.64, 11.05]	0.233

Unadjusted ORs. Reference levels: HSCL-25 anxiety score < 1.75 points, average SES

To test the second hypothesis, whether the strength of the association differs between male and female refugees, we consider the sex-stratified results as well as the interaction analysis. Comparing males to females with an average SES, the OR for symptoms of anxiety amounted to 0.26 (95% CI [0.22, 0.30]), and the OR for symptoms of depression amounted to 0.28 (95% CI [0.23, 0.32]) (Table 5), indicating that women are much more affected than men. A similar result could be seen when comparing depression scores in males and females with below average SES, while confidence intervals for comparisons of other SES strata were too wide to give informative results.

**Table 5:** ORs contrasting males and females across strata of SES

SES	HSCL-25 anxiety score		HSCL-25 depression score	
	OR [95% CI]	Adjusted *p* value	OR [95% CI]	adjusted *p* value
Below average	1.03 [0.23, 4.52]	1.000	0.26 [0.20, 0.34]	0.045
On average	0.26 [0.22, 0.30]	< 0.001	0.28 [0.23, 0.32]	< 0.001
Above average	0.73 [0.11, 5.03]	1.000	0.45 [0.17, 1.21]	1.000
Do not know/do not wish to answer	0.24 [0.16, 0.26]	0.320	0.36 [0.20, 0.67]	0.939

Reference levels: HSCL-25 anxiety/depression score < 1.75 points, female sex

### Sensitivity analyses

When all missing observations in the HSCL-25 anxiety score were set to no symptoms, i.e., values below 1.75, the resulting ORs were strongly reduced. All other sensitivity analyses support the described results of the main analysis. Tables with the detailed results of the sensitivity analyses can be found in the Additional file 3, Supplement 4.

### Correlation of SES and education variables

A weak positive correlation between SES and each of the education variables with Kendall’s Tau rank correlation coefficients for SES and years of education: 0.16, *p* < 0.001; SES and highest level of education: 0.15, *p* < 0.001 could be observed (for visual presentation, see Additional file 3: Supplement 3). Only three levels of SES (below/on/above average) were considered for these calculations. The two education variables showed a strong positive correlation with each other (Kendall’s Tau: 0.78,* p* < 0.001).

## Discussion

### Main findings

The aim of this study was to assess the association between pre-migration SES and clinically relevant levels of anxiety and depressive symptoms in Syrian refugees residing in Lebanon shortly after their migration as an example of migration from and to a low-middle income country (LMIC). We proposed two hypotheses: (1) the differences across strata of SES in general, and (2) whether the potential differences across strata of SES are modified by sex.

First, below average SES was associated with higher odds for clinically relevant levels of anxiety and depression when compared with average SES. Above average SES was also associated with increased odds compared with average SES, although the association does not reach statistical significance. Given the small sample size and imprecise estimates it is unclear whether this indicates a null-difference between the strata of above average and average SES.

Second, regarding the interaction between SES and sex, the analyses indicated that mental health differences by SES are less pronounced in females compared with males in the HSCL-25 anxiety score. Furthermore, among those with an average SES, males showed statistically significant lower odds than females for clinically relevant levels of anxiety or depressive symptoms. However, conclusions on sex differences remain incomplete. Due to the small number of males and of individuals with an above average SES in the sample, subgroup analysis yielded few statistically significant results.

### Comparison with existing literature

The study’s results were in line with the finding of Hollander et al. that lower education levels are associated with more mental health problems among Syrian refugees in Sweden. Additionally, Nissen et al. showed post-migration stressors like financial or social strain to be associated with poorer subjective well-being. Assuming that pre-migration SES continues to create socioeconomic differences in terms of financial or social affluence even after the flight, this finding is also in line with the observed difference between below average and average SES in the present study.

In terms of the interaction between SES and sex, our findings align with the study by Nissen et al. [5], which also found stronger differences due to financial or social strain in males compared with females, while overall subjective well-being was higher in males than in females. Furthermore, Bauer et al. reported that post-migration health satisfaction was similar across SES levels, and the range of health satisfaction was similar for males and females, although the general level of health satisfaction was slightly lower for females compared to males. However, the absence of a health gradient by SES level in that study could be attributed to the use of another variable, namely, health satisfaction, for measuring health. The difference might also in part be due to different effects of migration to Germany as a high-income country in Bauer’s study as opposed to Lebanon as a low/middle-income country in this study.

The alignment between our findings and the existing literature supports the robustness and validity of the associations identified in this study.

### Interpretability of findings

The exact mechanisms underlying the observed mental health differences by SES require further investigation. However, irrespective of specific causal pathways, the findings underscore the need for interventions targeted at individuals with below average pre-migration SES. Regardless of the causal direction, efforts to reduce either socioeconomic or mental health disparities likely also reduce the other [34].

Understanding SES as a multifaceted concept is crucial in interpreting the study's findings. SES showed weak monotonous positive correlations with both of the education variables in our study. It may therefore be assumed that study participants did not only consider their educational achievements when assessing their pre-migration SES. As a compound measure, SES encompasses education, income and occupation and, as we used a self-reported measure of SES, also captures socio-psychological effects and subjective perceptions of social position [15, 35].

Moreover, the study highlights the importance of considering sex and gender in understanding mental health disparities. Different factors, including biological sex (e.g., sex hormones) and gender (e.g., environmental factors such as the different number of stressors experienced by women and men, and differential susceptibility to stressors depending on the gender role), contribute to mental health differences [36, 37]. Some uncertainty remains regarding the sex variable used in this study. While the question to assess sex was posed with the options “male”, “female” and “do not want to answer”, and it was the clear impression among the data collectors that participants answered this according to (biological) sex, we cannot rule out that some participants may have answered according to gender.

### Implications for future interventions

Healthcare interventions have proven to be an effective measure to improve mental health also in those with lower SES, particularly when they are culturally adapted and include booster sessions to support long-term positive effects [38]. An effort to enhance healthcare seeking and receiving for this group would further contribute to successful treatment of mental health problems. Additionally, including measures targeting social and economic needs in healthcare might help reduce both health and socioeconomic differences as post-migration stressors, such as financial or social strain, may act as mediators in the relationship between SES and mental health outcomes [5]. Finally, clarity in the measurement of sex and/or gender variables is essential for exploring potential causes of observed differences to guide interventions aimed at promoting equality.

### Strengths and limitations

This study represents an important contribution to the scarce literature on social determinants of refugees’ mental health and raises awareness of the heterogeneity among a specific refugee population, both along the lines of socioeconomic status and sex. It thereby points out the need for diverse responses and calls for approaches that consider this diversity. It helps to identify the most vulnerable to mental ill-health who need to be the priority when designing interventions for the improvement of refugee mental health. Strengths of this study further include the use of a validated questionnaire to assess mental health parameters of the participants as well as assessment of the informative value of the subjective SES variable used.

One limitation consists in the cross-sectional study design without longitudinal data on either SES or mental health available and no confounder control in the analysis which makes causal interpretation of the results impossible. Another limitation is the moderate sample size and sparse-data problems in substrata, a problem often occurring in intersectionality-informed analyses Furthermore, while the complete-case and missing indicator methods used in the main analysis have the advantage of being easy to implement, they pose the risk of biased results if missing observations are not missing completely at random. Finally, it is not certain whether the widely used threshold of 1.75 for the HSCL-25 score is valid for the study population in this study. Two sensitivity analyses dealing with the latter two limitations by (1) different handling of missing data and (2) changed thresholds for the HSCL-25 variables yielded results that were similar to the main analysis, supporting the robustness of the general results of this study.

## Conclusions

High levels of anxiety and depressive symptoms on the HSCL-25 scale were observed in Syrian refugees in Lebanon, with overall levels among females higher than those among males. This underlines the importance of targeted interventions to the entire Syrian refugee population in Lebanon to improve their mental health status after the often-traumatic experience of forced migration. An association between SES and anxiety as well as between SES and depressive symptoms could be shown wherein individuals with a below average SES showed significantly increased symptoms in comparison to those with an average SES. The differences in mental health across strata of SES were more pronounced in males than in females.

For further research, it is therefore of interest to study mechanisms underlying this association with the aim to identify causal factors that can be addressed in interventions to treat or prevent adverse mental health outcomes. This might be, for example, varying degrees of health literacy to deal with mental challenges, or differences in health care access and utilization. Regardless of the causal direction of the underlying mechanisms, reducing socioeconomic and mental health disparities go hand in hand and more socioeconomic equality will often result in less mental health disparities and vice versa.

### Supplementary Information

**Additional file 1.** R code of statistical analysis.

**Additional file 2.** Report of statistical analysis results.

**Additional file 3: Supplement 1.** Discussion of missingness. **Supplement 2.** Baseline characteristics of participants included in the main analysis. **Supplement 3.** Examination of associations between SES and education variables. **Supplement 4.** Results of sensitivity analyses.

## Data Availability

The datasets generated and/or analyzed during the current study are not publicly available due to Danish data protection regulations but are available from AHE upon reasonable request.

## Abbreviations

ARCH study: Asylum seekers’ and refugees’ changing health study
CI: Confidence interval
FDP: Forcibly displaced person
HSCL-25: Hopkins symptom checklist-25
OR: Odds ratio
PTSD: Post-traumatic stress disorder
ref: Reference level
SES: Socioeconomic status
