# Supplementary material for: Association of pre-migration socioeconomic status and post-migration mental health in Syrian refugees in Lebanon: a descriptive sex-stratified cross-sectional analysis
Source: Glob Health Res Policy. 2024 Mar 4;9:9. doi: 10.1186/s41256-024-00347-0 (PMC10910804; doi:10.1186/s41256-024-00347-0)
Supplement: Supplementary file 2 — Additional file 2. Report of statistical analysis results. [file 41256_2024_347_MOESM2_ESM.html]

Assoc. of Pre-Migration Socioeconomic Status and Post-Migration Mental Health


Code 

- Show All Code
- Hide All Code

# Assoc. of Pre-Migration Socioeconomic Status and Post-Migration Mental Health

#### Saskia Lange

#### 2023

```
sessionInfo()
```

```
## R version 4.3.2 (2023-10-31)
## Platform: x86_64-pc-linux-gnu (64-bit)
## Running under: Ubuntu 22.04.3 LTS
## 
## Matrix products: default
## BLAS:   /usr/lib/x86_64-linux-gnu/blas/libblas.so.3.10.0 
## LAPACK: /usr/lib/x86_64-linux-gnu/lapack/liblapack.so.3.10.0
## 
## locale:
##  [1] LC_CTYPE=en_GB.UTF-8       LC_NUMERIC=C              
##  [3] LC_TIME=en_GB.UTF-8        LC_COLLATE=en_GB.UTF-8    
##  [5] LC_MONETARY=en_GB.UTF-8    LC_MESSAGES=en_GB.UTF-8   
##  [7] LC_PAPER=en_GB.UTF-8       LC_NAME=C                 
##  [9] LC_ADDRESS=C               LC_TELEPHONE=C            
## [11] LC_MEASUREMENT=en_GB.UTF-8 LC_IDENTIFICATION=C       
## 
## time zone: Europe/Copenhagen
## tzcode source: system (glibc)
## 
## attached base packages:
## [1] splines   stats4    stats     graphics  grDevices utils     datasets 
## [8] methods   base     
## 
## other attached packages:
##  [1] multtest_2.58.0     Biobase_2.62.0      BiocGenerics_0.48.1
##  [4] BiocManager_1.30.22 effects_4.2-2       carData_3.0-5      
##  [7] expss_0.11.6        maditr_0.8.3        mice_3.14.0        
## [10] epiDisplay_3.5.0.2  nnet_7.3-19         MASS_7.3-60        
## [13] survival_3.5-7      foreign_0.8-85      mutoss_0.1-13      
## [16] mvtnorm_1.1-3       emmeans_1.8.9       naniar_1.0.0       
## [19] table1_1.4.3        VGAM_1.1-6          skimr_2.1.5        
## [22] forcats_0.5.1       stringr_1.4.0       dplyr_1.0.8        
## [25] purrr_0.3.4         readr_2.1.2         tidyr_1.2.0        
## [28] tibble_3.1.6        ggplot2_3.4.4       tidyverse_1.3.1    
## 
## loaded via a namespace (and not attached):
##  [1] DBI_1.1.2          sandwich_3.0-1     readxl_1.3.1       rlang_1.1.2       
##  [5] magrittr_2.0.2     multcomp_1.4-18    matrixStats_0.61.0 compiler_4.3.2    
##  [9] vctrs_0.6.4        rvest_1.0.2        pkgconfig_2.0.3    crayon_1.5.0      
## [13] fastmap_1.1.0      backports_1.4.1    dbplyr_2.1.1       ellipsis_0.3.2    
## [17] utf8_1.2.2         rmarkdown_2.25     tzdb_0.2.0         nloptr_2.0.0      
## [21] haven_2.4.3        visdat_0.6.0       xfun_0.41          reprex_2.0.1      
## [25] cachem_1.0.6       jsonlite_1.7.3     broom_0.7.12       R6_2.5.1          
## [29] bslib_0.5.1        stringi_1.7.6      boot_1.3-28        lubridate_1.8.0   
## [33] jquerylib_0.1.4    cellranger_1.1.0   estimability_1.4.1 Rcpp_1.0.8        
## [37] assertthat_0.2.1   knitr_1.37         modelr_0.1.8       zoo_1.8-9         
## [41] base64enc_0.1-3    Matrix_1.6-2       tidyselect_1.1.2   rstudioapi_0.13   
## [45] yaml_2.3.5         codetools_0.2-19   lattice_0.22-5     withr_2.5.2       
## [49] coda_0.19-4        evaluate_0.15      survey_4.2-1       xml2_1.3.3        
## [53] pillar_1.7.0       checkmate_2.0.0    insight_0.19.6     generics_0.1.2    
## [57] hms_1.1.1          munsell_0.5.0      scales_1.2.1       minqa_1.2.4       
## [61] xtable_1.8-4       glue_1.6.1         tools_4.3.2        data.table_1.14.2 
## [65] lme4_1.1-28        fs_1.5.2           grid_4.3.2         plotrix_3.8-2     
## [69] mitools_2.4        colorspace_2.0-2   nlme_3.1-163       repr_1.1.6        
## [73] htmlTable_2.4.0    Formula_1.2-4      cli_3.6.1          fansi_1.0.2       
## [77] gtable_0.3.0       sass_0.4.0         digest_0.6.29      TH.data_1.1-0     
## [81] htmlwidgets_1.5.4  htmltools_0.5.7    lifecycle_1.0.4    httr_1.4.2
```

## Skimming Data

```
skimr::skim(data)
```

Data summary

|  |  |
| --- | --- |
| Name | data |
| Number of rows | 599 |
| Number of columns | 12 |
| \_\_\_\_\_\_\_\_\_\_\_\_\_\_\_\_\_\_\_\_\_\_\_ |  |
| Column type frequency: |  |
| character | 1 |
| factor | 5 |
| numeric | 6 |
| \_\_\_\_\_\_\_\_\_\_\_\_\_\_\_\_\_\_\_\_\_\_\_\_ |  |
| Group variables | None |

**Variable type: character**

| skim\_variable | n\_missing | complete\_rate | min | max | empty | n\_unique | whitespace |
| --- | --- | --- | --- | --- | --- | --- | --- |
| sex | 0 | 1 | 4 | 7 | 0 | 3 | 0 |

**Variable type: factor**

| skim\_variable | n\_missing | complete\_rate | ordered | n\_unique | top\_counts |
| --- | --- | --- | --- | --- | --- |
| highest\_educ | 27 | 0.95 | FALSE | 4 | Pri: 246, No : 188, Hig: 109, Hig: 29 |
| ses | 61 | 0.90 | FALSE | 3 | On : 365, Bel: 137, Do : 36 |
| hscl\_anx\_dich | 54 | 0.91 | FALSE | 2 | yes: 434, no: 111 |
| hscl\_dep\_dich | 120 | 0.80 | FALSE | 2 | yes: 380, no: 99 |
| hscl\_tot\_dich | 129 | 0.78 | FALSE | 2 | yes: 388, no: 82 |

**Variable type: numeric**

| skim\_variable | n\_missing | complete\_rate | mean | sd | p0 | p25 | p50 | p75 | p100 | hist |
| --- | --- | --- | --- | --- | --- | --- | --- | --- | --- | --- |
| age | 58 | 0.90 | 37.39 | 13.42 | 18 | 27.00 | 35.00 | 47.00 | 90 | ▇▆▃▁▁ |
| years\_education | 195 | 0.67 | 6.63 | 4.54 | 0 | 4.00 | 6.00 | 9.00 | 21 | ▆▇▆▁▁ |
| hscl\_anx\_score | 54 | 0.91 | 2.41 | 0.72 | 1 | 1.90 | 2.40 | 2.90 | 4 | ▅▆▇▆▂ |
| hscl\_depres\_score | 120 | 0.80 | 2.33 | 0.67 | 1 | 1.87 | 2.33 | 2.77 | 4 | ▅▇▇▅▂ |
| months\_in\_host\_country | 0 | 1.00 | 7.90 | 1.90 | 1 | 7.00 | 8.00 | 9.00 | 12 | ▁▂▇▇▅ |
| hscl\_total | 129 | 0.78 | 2.37 | 0.64 | 1 | 1.92 | 2.39 | 2.81 | 4 | ▃▆▇▅▂ |

## Descriptive tables

Table 1

```
tab1 <- table1::table1(~ age + months_in_host_country + ses + highest_educ + years_education + hscl_anx_score + hscl_anx_dich + hscl_depres_score + hscl_dep_dich | sex, data = data)
tab1
```

|  | Female (N=408) | Male (N=153) | missing (N=38) | Overall (N=599) |
| --- | --- | --- | --- | --- |
| age |  |  |  |  |
| Mean (SD) | 36.5 (12.7) | 39.7 (15.0) | 36.0 (8.49) | 37.4 (13.4) |
| Median [Min, Max] | 34.0 [18.0, 71.0] | 38.0 [18.0, 90.0] | 35.0 [25.0, 53.0] | 35.0 [18.0, 90.0] |
| Missing | 27 (6.6%) | 2 (1.3%) | 29 (76.3%) | 58 (9.7%) |
| months\_in\_host\_country |  |  |  |  |
| Mean (SD) | 7.83 (1.93) | 8.01 (1.81) | 8.26 (2.00) | 7.90 (1.90) |
| Median [Min, Max] | 8.00 [1.00, 12.0] | 8.00 [4.00, 12.0] | 8.00 [5.00, 12.0] | 8.00 [1.00, 12.0] |
| ses |  |  |  |  |
| Below average | 90 (22.1%) | 43 (28.1%) | 4 (10.5%) | 137 (22.9%) |
| On or above average | 263 (64.5%) | 96 (62.7%) | 6 (15.8%) | 365 (60.9%) |
| Do not know/do not wish to answer | 30 (7.4%) | 6 (3.9%) | 0 (0%) | 36 (6.0%) |
| Missing | 25 (6.1%) | 8 (5.2%) | 28 (73.7%) | 61 (10.2%) |
| highest\_educ |  |  |  |  |
| No education | 131 (32.1%) | 46 (30.1%) | 11 (28.9%) | 188 (31.4%) |
| Primary school | 172 (42.2%) | 73 (47.7%) | 1 (2.6%) | 246 (41.1%) |
| High school | 82 (20.1%) | 25 (16.3%) | 2 (5.3%) | 109 (18.2%) |
| Higher | 20 (4.9%) | 9 (5.9%) | 0 (0%) | 29 (4.8%) |
| Missing | 3 (0.7%) | 0 (0%) | 24 (63.2%) | 27 (4.5%) |
| years\_education |  |  |  |  |
| Mean (SD) | 6.44 (4.40) | 7.07 (4.86) | 9.00 (NA) | 6.63 (4.54) |
| Median [Min, Max] | 6.00 [0, 20.0] | 6.00 [0, 21.0] | 9.00 [9.00, 9.00] | 6.00 [0, 21.0] |
| Missing | 121 (29.7%) | 37 (24.2%) | 37 (97.4%) | 195 (32.6%) |
| hscl\_anx\_score |  |  |  |  |
| Mean (SD) | 2.52 (0.706) | 2.12 (0.717) | 2.24 (0.389) | 2.41 (0.724) |
| Median [Min, Max] | 2.50 [1.00, 4.00] | 2.10 [1.00, 3.80] | 2.20 [1.50, 2.80] | 2.40 [1.00, 4.00] |
| Missing | 25 (6.1%) | 6 (3.9%) | 23 (60.5%) | 54 (9.0%) |
| hscl\_anx\_dich |  |  |  |  |
| no | 60 (14.7%) | 49 (32.0%) | 2 (5.3%) | 111 (18.5%) |
| yes | 323 (79.2%) | 98 (64.1%) | 13 (34.2%) | 434 (72.5%) |
| Missing | 25 (6.1%) | 6 (3.9%) | 23 (60.5%) | 54 (9.0%) |
| hscl\_depres\_score |  |  |  |  |
| Mean (SD) | 2.45 (0.646) | 2.04 (0.646) | 2.06 (0.245) | 2.33 (0.665) |
| Median [Min, Max] | 2.40 [1.00, 4.00] | 2.07 [1.00, 3.67] | 2.07 [1.47, 2.47] | 2.33 [1.00, 4.00] |
| Missing | 77 (18.9%) | 18 (11.8%) | 25 (65.8%) | 120 (20.0%) |
| hscl\_dep\_dich |  |  |  |  |
| no | 48 (11.8%) | 50 (32.7%) | 1 (2.6%) | 99 (16.5%) |
| yes | 283 (69.4%) | 85 (55.6%) | 12 (31.6%) | 380 (63.4%) |
| Missing | 77 (18.9%) | 18 (11.8%) | 25 (65.8%) | 120 (20.0%) |

## Correlation of SES and Education variables

### ses and years\_education

```
data %>% 
  tidyr::drop_na(ses, years_education) %>% 
  ggplot2::ggplot(mapping = ggplot2::aes(x=ses, y=years_education)) +
  ggplot2::geom_boxplot() +
  ggplot2::geom_jitter(alpha=0.2) +
  ggplot2::labs(x = "SES", y = "years of education") +
  ggplot2::scale_x_discrete(labels = c("Below average", "On average", "Above average", "Do not know/ \n do not wish to answer"))
```

kendall’s tau: (with three levels of ses: below/on/above average)

```
data_cor <- data %>% 
  dplyr::filter(ses == "Below average" | ses == "On average" | ses == "Above average") %>% 
  dplyr::select(ses, years_education, highest_educ) %>% 
  dplyr::mutate(ses = as.numeric(ses, levels = c("Below average", "On average", "Above average"))) %>% 
  dplyr::mutate(highest_educ = as.numeric(highest_educ, levels = c("No education", "Primary school", "High school", "Higher")))
# kendall.tau(data_cor$ses, data_cor$years_education)

cor.test(data_cor$ses,data_cor$years_education, method = "kendall")
```

```
## 
##  Kendall's rank correlation tau
## 
## data:  data_cor$ses and data_cor$years_education
## T = NA, p-value = NA
## alternative hypothesis: true tau is not equal to 0
## sample estimates:
## tau 
##  NA
```

```
# cor(data_cor, method = "kendall", use = "pairwise")
```

### ses and highest\_educ

```
#highest_educ: absolute numbers
data %>% 
  tidyr::drop_na(ses, highest_educ) %>% 
  ggplot2::ggplot(mapping = ggplot2::aes(x = ses, y = highest_educ)) +
  ggplot2::geom_count() +
  ggplot2::labs(x = "SES", y = "highest education level") +
  ggplot2::scale_x_discrete(labels = c("Below average", "On average", "Above average", "Do not know/ \n do not wish to answer"))
```

```
#highest_educ: proportions
data %>% 
  tidyr::drop_na(ses, highest_educ) %>% 
  ggplot2::ggplot(mapping = ggplot2::aes(x = ses, y = highest_educ)) +
  ggplot2::geom_count(ggplot2::aes(size=ggplot2::after_stat(prop), group = ses)) +
  ggplot2::labs(x = "SES", y = "highest education level", size = "Proportion") +
  ggplot2::scale_x_discrete(labels = c("Below average", "On average", "Above average", "Do not know/ \n do not wish to answer"))
```

kendall’s tau: (with three levels of ses: below/on/above average)

```
# kendall.tau(data_cor$ses, data_cor$highest_educ)
cor.test(x=data_cor$ses, data_cor$highest_educ, method = "kendall", use = "pairwise")
```

```
## 
##  Kendall's rank correlation tau
## 
## data:  data_cor$ses and data_cor$highest_educ
## T = NA, p-value = NA
## alternative hypothesis: true tau is not equal to 0
## sample estimates:
## tau 
##  NA
```

### years\_education and highest\_educ

```
#education variables
data %>% 
  tidyr::drop_na(years_education, highest_educ) %>% 
  ggplot2::ggplot(mapping = ggplot2::aes(x = highest_educ, y = years_education)) +
  ggplot2::geom_boxplot()+
  ggplot2::geom_jitter(alpha=0.2) +
  ggplot2::labs(x = "highest education level", y = "years of education")
```

kendall’s tau:

```
data_compl_ed <- data %>% 
  tidyr::drop_na(highest_educ, years_education) %>% 
  dplyr::mutate(highest_educ = as.numeric(highest_educ, levels = c("No education", "Primary school", "High school", "Higher")))

# kendall.tau(x=data_compl_ed$years_education, y=data_compl_ed$highest_educ)

cor.test(data_cor$years_education, data_cor$highest_educ, method = "kendall")
```

```
## 
##  Kendall's rank correlation tau
## 
## data:  data_cor$years_education and data_cor$highest_educ
## z = 9.6958, p-value < 2.2e-16
## alternative hypothesis: true tau is not equal to 0
## sample estimates:
##       tau 
## 0.8252139
```

```
## Correlation of HSCL-25 scores
# ggplot(data, mapping = aes(x = hscl_anx_score, y = hscl_depres_score)) +
#   geom_point() +
#   geom_smooth()
```

## Exploring Missingness patterns

```
data <- data %>% 
  dplyr::mutate(sex = dplyr::na_if(sex, "missing")) %>% 
  dplyr::mutate(sex = as.factor(sex))

data %>% 
  is.na() %>% 
  colSums()
```

```
##                    age        years_education                    sex 
##                     58                    195                     38 
##           highest_educ                    ses         hscl_anx_score 
##                     27                     61                     54 
##      hscl_depres_score months_in_host_country          hscl_anx_dich 
##                    120                      0                     54 
##          hscl_dep_dich             hscl_total          hscl_tot_dich 
##                    120                    129                    129
```

```
data %>% 
  dplyr::select(age, sex, years_education, highest_educ, ses, hscl_anx_score, hscl_depres_score) %>% 
  naniar::gg_miss_upset(nsets = 7)
```

```
data %>% 
   dplyr::select(age, sex, years_education, highest_educ, ses, hscl_anx_score, hscl_depres_score) %>% 
naniar::gg_miss_fct(x = ., fct = ses) + 
  ggplot2::labs(x = "SES", y = "Variable", color = "% Missing") +
  ggplot2::scale_x_discrete(labels = c("Below average", "On average", "Above average", "Do not know/ \n do not wish to answer"))
```

```
data %>% 
  dplyr::select(age, sex, years_education, highest_educ, ses, hscl_anx_score, hscl_depres_score) %>% 
naniar::gg_miss_fct(x = ., fct = sex) +
  ggplot2::labs(x = "Sex", y = "Variable", color = "% Missing") +
  ggplot2::scale_x_discrete(labels = c("Female", "Male", "NA"))
```

```
data %>% 
  dplyr::select(age, sex, years_education, highest_educ, ses, hscl_anx_score, hscl_depres_score) %>% 
naniar::gg_miss_fct(x = ., fct = highest_educ) + 
  ggplot2::labs(x = "Highest education level", y = "Variable", color = "% Missing") +
  ggplot2::scale_x_discrete(labels = c("No education", "Primary school", "High School", "Higher", "NA"))
```

```
data %>% 
  dplyr::select(sex, years_education, highest_educ, ses, hscl_anx_score, hscl_depres_score) %>%
 naniar:: miss_case_table()
```

```
## # A tibble: 7 × 3
##   n_miss_in_case n_cases pct_cases
##            <int>   <int>     <dbl>
## 1              0     337    56.3  
## 2              1     157    26.2  
## 3              2      54     9.02 
## 4              3      23     3.84 
## 5              4       2     0.334
## 6              5       3     0.501
## 7              6      23     3.84
```

# Analysis

## Imputed SES data

```
## data set prep
data_complete <- data %>% 
  dplyr::mutate(ses = expss::if_na(ses, "Do not know/do not wish to answer")) %>% 
  dplyr::filter(complete.cases(sex, ses, hscl_anx_score, hscl_depres_score))

data_complete <- data_complete %>% 
  dplyr::mutate(hscl_anx_dich = dplyr::case_when(
    hscl_anx_score < 1.75 ~ "no",
    hscl_anx_score >= 1.75 ~ "yes"
  )) %>% 
  dplyr::mutate(hscl_anx_dich = as.factor(hscl_anx_dich))

data_complete <- data_complete %>% 
  dplyr::mutate(hscl_dep_dich = dplyr::case_when(
    hscl_depres_score < 1.75 ~ "no",
    hscl_depres_score >= 1.75 ~ "yes"
  )) %>% 
  dplyr::mutate(hscl_dep_dich = as.factor(hscl_dep_dich))

## prep for anaylsis - setting reference levels
data_complete$ses <- factor(data_complete$ses, ordered = F)
data_complete$ses <- relevel(data_complete$ses, ref = "On or above average")
data_complete$hscl_anx_dich <- relevel(data_complete$hscl_anx_dich, ref = "no")
data_complete$hscl_dep_dich <- relevel(data_complete$hscl_dep_dich, ref = "no")
```

Table 1

```
tab1_complete <- table1::table1(~ age + months_in_host_country + ses + highest_educ + years_education + hscl_anx_score + hscl_anx_dich + hscl_depres_score + hscl_dep_dich | sex, data = data_complete)
tab1_complete
```

|  | Female (N=322) | Male (N=135) | Overall (N=457) |
| --- | --- | --- | --- |
| age |  |  |  |
| Mean (SD) | 36.3 (12.8) | 39.1 (13.6) | 37.2 (13.1) |
| Median [Min, Max] | 33.5 [18.0, 71.0] | 38.0 [18.0, 72.0] | 35.0 [18.0, 72.0] |
| Missing | 20 (6.2%) | 1 (0.7%) | 21 (4.6%) |
| months\_in\_host\_country |  |  |  |
| Mean (SD) | 7.91 (1.87) | 8.00 (1.82) | 7.94 (1.85) |
| Median [Min, Max] | 8.00 [2.00, 12.0] | 8.00 [4.00, 12.0] | 8.00 [2.00, 12.0] |
| ses |  |  |  |
| On or above average | 207 (64.3%) | 83 (61.5%) | 290 (63.5%) |
| Below average | 78 (24.2%) | 40 (29.6%) | 118 (25.8%) |
| Do not know/do not wish to answer | 37 (11.5%) | 12 (8.9%) | 49 (10.7%) |
| highest\_educ |  |  |  |
| No education | 101 (31.4%) | 40 (29.6%) | 141 (30.9%) |
| Primary school | 138 (42.9%) | 66 (48.9%) | 204 (44.6%) |
| High school | 67 (20.8%) | 20 (14.8%) | 87 (19.0%) |
| Higher | 16 (5.0%) | 9 (6.7%) | 25 (5.5%) |
| years\_education |  |  |  |
| Mean (SD) | 6.31 (4.42) | 7.04 (4.95) | 6.53 (4.59) |
| Median [Min, Max] | 6.00 [0, 20.0] | 6.00 [0, 21.0] | 6.00 [0, 21.0] |
| Missing | 81 (25.2%) | 31 (23.0%) | 112 (24.5%) |
| hscl\_anx\_score |  |  |  |
| Mean (SD) | 2.54 (0.698) | 2.14 (0.723) | 2.42 (0.728) |
| Median [Min, Max] | 2.50 [1.00, 4.00] | 2.10 [1.00, 3.80] | 2.40 [1.00, 4.00] |
| hscl\_anx\_dich |  |  |  |
| no | 47 (14.6%) | 44 (32.6%) | 91 (19.9%) |
| yes | 275 (85.4%) | 91 (67.4%) | 366 (80.1%) |
| hscl\_depres\_score |  |  |  |
| Mean (SD) | 2.44 (0.643) | 2.04 (0.646) | 2.32 (0.668) |
| Median [Min, Max] | 2.40 [1.00, 4.00] | 2.07 [1.00, 3.67] | 2.33 [1.00, 4.00] |
| hscl\_dep\_dich |  |  |  |
| no | 48 (14.9%) | 50 (37.0%) | 98 (21.4%) |
| yes | 274 (85.1%) | 85 (63.0%) | 359 (78.6%) |

### SES and hscl\_anx

#### Total

```
model_comp1 <- glm(hscl_anx_dich ~ ses, family = "binomial", data = data_complete)
summary(model_comp1)
```

```
## 
## Call:
## glm(formula = hscl_anx_dich ~ ses, family = "binomial", data = data_complete)
## 
## Coefficients:
##                                      Estimate Std. Error z value Pr(>|z|)    
## (Intercept)                            1.0712     0.1347   7.953 1.82e-15 ***
## sesBelow average                       1.4229     0.3721   3.824 0.000131 ***
## sesDo not know/do not wish to answer   0.5629     0.4093   1.375 0.169042    
## ---
## Signif. codes:  0 '***' 0.001 '**' 0.01 '*' 0.05 '.' 0.1 ' ' 1
## 
## (Dispersion parameter for binomial family taken to be 1)
## 
##     Null deviance: 456.26  on 456  degrees of freedom
## Residual deviance: 436.64  on 454  degrees of freedom
## AIC: 442.64
## 
## Number of Fisher Scoring iterations: 5
```

```
exp(coefficients(model_comp1))
```

```
##                          (Intercept)                     sesBelow average 
##                             2.918919                             4.149177 
## sesDo not know/do not wish to answer 
##                             1.755787
```

```
exp(confint(model_comp1))
```

```
##                                          2.5 %   97.5 %
## (Intercept)                          2.2535421 3.824142
## sesBelow average                     2.1007544 9.181383
## sesDo not know/do not wish to answer 0.8257479 4.191790
```

```
mod1 <- lm(hscl_anx_score~ses, data = data_complete)
anova(mod1)
```

```
## Analysis of Variance Table
## 
## Response: hscl_anx_score
##            Df  Sum Sq Mean Sq F value  Pr(>F)  
## ses         2   4.169 2.08444   3.988 0.01919 *
## Residuals 454 237.298 0.52268                  
## ---
## Signif. codes:  0 '***' 0.001 '**' 0.01 '*' 0.05 '.' 0.1 ' ' 1
```

#### Males

```
data_completem <- data_complete %>% 
  dplyr::filter(sex == "Male")

model_comp1m <- glm(hscl_anx_dich ~ ses, family = "binomial", data = data_completem)
summary(model_comp1m)
```

```
## 
## Call:
## glm(formula = hscl_anx_dich ~ ses, family = "binomial", data = data_completem)
## 
## Coefficients:
##                                      Estimate Std. Error z value Pr(>|z|)    
## (Intercept)                            0.2177     0.2208   0.986 0.324168    
## sesBelow average                       2.2946     0.6396   3.587 0.000334 ***
## sesDo not know/do not wish to answer   0.4754     0.6510   0.730 0.465190    
## ---
## Signif. codes:  0 '***' 0.001 '**' 0.01 '*' 0.05 '.' 0.1 ' ' 1
## 
## (Dispersion parameter for binomial family taken to be 1)
## 
##     Null deviance: 170.44  on 134  degrees of freedom
## Residual deviance: 150.67  on 132  degrees of freedom
## AIC: 156.67
## 
## Number of Fisher Scoring iterations: 5
```

```
exp(coefficients(model_comp1m))
```

```
##                          (Intercept)                     sesBelow average 
##                             1.243243                             9.920290 
## sesDo not know/do not wish to answer 
##                             1.608696
```

```
exp(confint(model_comp1m))
```

```
##                                          2.5 %    97.5 %
## (Intercept)                          0.8078365  1.926405
## sesBelow average                     3.2488901 43.374844
## sesDo not know/do not wish to answer 0.4679147  6.412596
```

```
mod1m <- lm(hscl_anx_score~ses, data = data_completem)
anova(mod1m)
```

```
## Analysis of Variance Table
## 
## Response: hscl_anx_score
##            Df Sum Sq Mean Sq F value  Pr(>F)  
## ses         2  4.566 2.28276  4.5991 0.01173 *
## Residuals 132 65.518 0.49635                  
## ---
## Signif. codes:  0 '***' 0.001 '**' 0.01 '*' 0.05 '.' 0.1 ' ' 1
```

#### Females

```
data_completef <- data_complete %>% 
  dplyr::filter(sex == "Female")

model_comp1f <- glm(hscl_anx_dich ~ ses, family = "binomial", data = data_completef)
summary(model_comp1f)
```

```
## 
## Call:
## glm(formula = hscl_anx_dich ~ ses, family = "binomial", data = data_completef)
## 
## Coefficients:
##                                      Estimate Std. Error z value Pr(>|z|)    
## (Intercept)                            1.5249     0.1814   8.406   <2e-16 ***
## sesBelow average                       0.9600     0.4620   2.078   0.0377 *  
## sesDo not know/do not wish to answer   0.5853     0.5597   1.046   0.2956    
## ---
## Signif. codes:  0 '***' 0.001 '**' 0.01 '*' 0.05 '.' 0.1 ' ' 1
## 
## (Dispersion parameter for binomial family taken to be 1)
## 
##     Null deviance: 267.67  on 321  degrees of freedom
## Residual deviance: 262.02  on 319  degrees of freedom
## AIC: 268.02
## 
## Number of Fisher Scoring iterations: 5
```

```
exp(coefficients(model_comp1f))
```

```
##                          (Intercept)                     sesBelow average 
##                             4.594595                             2.611765 
## sesDo not know/do not wish to answer 
##                             1.795588
```

```
exp(confint(model_comp1f))
```

```
##                                          2.5 %   97.5 %
## (Intercept)                          3.2603873 6.653140
## sesBelow average                     1.1293581 7.123744
## sesDo not know/do not wish to answer 0.6628903 6.289306
```

```
mod1f <- lm(hscl_anx_score~ses, data = data_completef)
anova(mod1f)
```

```
## Analysis of Variance Table
## 
## Response: hscl_anx_score
##            Df  Sum Sq Mean Sq F value Pr(>F)
## ses         2   1.397 0.69856  1.4386 0.2388
## Residuals 319 154.903 0.48559
```

### SES and hscl\_depres

#### Total

```
model_comp2 <- glm(hscl_dep_dich ~ ses, family = "binomial", data = data_complete)
summary(model_comp2)
```

```
## 
## Call:
## glm(formula = hscl_dep_dich ~ ses, family = "binomial", data = data_complete)
## 
## Coefficients:
##                                      Estimate Std. Error z value Pr(>|z|)    
## (Intercept)                            1.0894     0.1353   8.052 8.16e-16 ***
## sesBelow average                       0.6254     0.2896   2.159   0.0308 *  
## sesDo not know/do not wish to answer   0.7023     0.4301   1.633   0.1025    
## ---
## Signif. codes:  0 '***' 0.001 '**' 0.01 '*' 0.05 '.' 0.1 ' ' 1
## 
## (Dispersion parameter for binomial family taken to be 1)
## 
##     Null deviance: 475.08  on 456  degrees of freedom
## Residual deviance: 468.23  on 454  degrees of freedom
## AIC: 474.23
## 
## Number of Fisher Scoring iterations: 4
```

```
exp(coefficients(model_comp2))
```

```
##                          (Intercept)                     sesBelow average 
##                             2.972603                             1.868920 
## sesDo not know/do not wish to answer 
##                             2.018433
```

```
exp(confint(model_comp2))
```

```
##                                          2.5 %   97.5 %
## (Intercept)                          2.2925507 3.899649
## sesBelow average                     1.0796396 3.379070
## sesDo not know/do not wish to answer 0.9200351 5.088539
```

```
mod2 <- lm(hscl_depres_score~ses, data = data_complete)
anova(mod2)
```

```
## Analysis of Variance Table
## 
## Response: hscl_depres_score
##            Df Sum Sq Mean Sq F value  Pr(>F)  
## ses         2   3.27  1.6348  3.7078 0.02528 *
## Residuals 454 200.17  0.4409                  
## ---
## Signif. codes:  0 '***' 0.001 '**' 0.01 '*' 0.05 '.' 0.1 ' ' 1
```

#### Males

```
model_comp2m <- glm(hscl_dep_dich ~ ses, family = "binomial", data = data_completem)
summary(model_comp2m)
```

```
## 
## Call:
## glm(formula = hscl_dep_dich ~ ses, family = "binomial", data = data_completem)
## 
## Coefficients:
##                                      Estimate Std. Error z value Pr(>|z|)  
## (Intercept)                            0.2666     0.2215   1.204   0.2287  
## sesBelow average                       0.7028     0.4177   1.683   0.0925 .
## sesDo not know/do not wish to answer   0.8320     0.7025   1.184   0.2363  
## ---
## Signif. codes:  0 '***' 0.001 '**' 0.01 '*' 0.05 '.' 0.1 ' ' 1
## 
## (Dispersion parameter for binomial family taken to be 1)
## 
##     Null deviance: 177.97  on 134  degrees of freedom
## Residual deviance: 174.15  on 132  degrees of freedom
## AIC: 180.15
## 
## Number of Fisher Scoring iterations: 4
```

```
exp(coefficients(model_comp2m))
```

```
##                          (Intercept)                     sesBelow average 
##                             1.305556                             2.019342 
## sesDo not know/do not wish to answer 
##                             2.297872
```

```
exp(confint(model_comp2m))
```

```
##                                          2.5 %    97.5 %
## (Intercept)                          0.8478901  2.027164
## sesBelow average                     0.9083158  4.722601
## sesDo not know/do not wish to answer 0.6331963 10.915245
```

```
mod2m <- lm(hscl_depres_score~ses, data = data_completem)
anova(mod2m)
```

```
## Analysis of Variance Table
## 
## Response: hscl_depres_score
##            Df Sum Sq Mean Sq F value Pr(>F)  
## ses         2  1.956 0.97787  2.3942 0.0952 .
## Residuals 132 53.914 0.40844                 
## ---
## Signif. codes:  0 '***' 0.001 '**' 0.01 '*' 0.05 '.' 0.1 ' ' 1
```

#### Females

```
model_comp2f <- glm(hscl_dep_dich ~ ses, family = "binomial", data = data_completef)
summary(model_comp2f)
```

```
## 
## Call:
## glm(formula = hscl_dep_dich ~ ses, family = "binomial", data = data_completef)
## 
## Coefficients:
##                                      Estimate Std. Error z value Pr(>|z|)    
## (Intercept)                            1.5249     0.1814   8.406   <2e-16 ***
## sesBelow average                       0.7919     0.4356   1.818   0.0691 .  
## sesDo not know/do not wish to answer   0.5853     0.5596   1.046   0.2956    
## ---
## Signif. codes:  0 '***' 0.001 '**' 0.01 '*' 0.05 '.' 0.1 ' ' 1
## 
## (Dispersion parameter for binomial family taken to be 1)
## 
##     Null deviance: 271.18  on 321  degrees of freedom
## Residual deviance: 266.82  on 319  degrees of freedom
## AIC: 272.82
## 
## Number of Fisher Scoring iterations: 4
```

```
exp(coefficients(model_comp2f))
```

```
##                          (Intercept)                     sesBelow average 
##                             4.594595                             2.207563 
## sesDo not know/do not wish to answer 
##                             1.795588
```

```
exp(confint(model_comp2f))
```

```
##                                          2.5 %   97.5 %
## (Intercept)                          3.2603873 6.653140
## sesBelow average                     0.9928868 5.614522
## sesDo not know/do not wish to answer 0.6628903 6.289307
```

```
mod2f <- lm(hscl_depres_score~ses, data = data_completef)
anova(mod2f)
```

```
## Analysis of Variance Table
## 
## Response: hscl_depres_score
##            Df  Sum Sq Mean Sq F value  Pr(>F)  
## ses         2   2.067  1.0334  2.5256 0.08161 .
## Residuals 319 130.534  0.4092                  
## ---
## Signif. codes:  0 '***' 0.001 '**' 0.01 '*' 0.05 '.' 0.1 ' ' 1
```

### interaction term ses\*sex

#### anxiety

```
#model: interaction ses*sex
model_interact1 <- glm(hscl_anx_dich ~ ses*sex, family = "binomial", data = data_complete)
summary(model_interact1)
```

```
## 
## Call:
## glm(formula = hscl_anx_dich ~ ses * sex, family = "binomial", 
##     data = data_complete)
## 
## Coefficients:
##                                              Estimate Std. Error z value
## (Intercept)                                    1.5249     0.1814   8.406
## sesBelow average                               0.9600     0.4620   2.078
## sesDo not know/do not wish to answer           0.5853     0.5597   1.046
## sexMale                                       -1.3072     0.2858  -4.574
## sesBelow average:sexMale                       1.3346     0.7890   1.691
## sesDo not know/do not wish to answer:sexMale  -0.1099     0.8585  -0.128
##                                              Pr(>|z|)    
## (Intercept)                                   < 2e-16 ***
## sesBelow average                               0.0377 *  
## sesDo not know/do not wish to answer           0.2956    
## sexMale                                      4.79e-06 ***
## sesBelow average:sexMale                       0.0908 .  
## sesDo not know/do not wish to answer:sexMale   0.8981    
## ---
## Signif. codes:  0 '***' 0.001 '**' 0.01 '*' 0.05 '.' 0.1 ' ' 1
## 
## (Dispersion parameter for binomial family taken to be 1)
## 
##     Null deviance: 456.26  on 456  degrees of freedom
## Residual deviance: 412.69  on 451  degrees of freedom
## AIC: 424.69
## 
## Number of Fisher Scoring iterations: 5
```

```
exp(coefficients(model_interact1))
```

```
##                                  (Intercept) 
##                                    4.5945946 
##                             sesBelow average 
##                                    2.6117647 
##         sesDo not know/do not wish to answer 
##                                    1.7955882 
##                                      sexMale 
##                                    0.2705882 
##                     sesBelow average:sexMale 
##                                    3.7983092 
## sesDo not know/do not wish to answer:sexMale 
##                                    0.8959157
```

```
exp(confint(model_interact1))
```

```
##                                                  2.5 %     97.5 %
## (Intercept)                                  3.2603873  6.6531404
## sesBelow average                             1.1293581  7.1237438
## sesDo not know/do not wish to answer         0.6628903  6.2893059
## sexMale                                      0.1538128  0.4727804
## sesBelow average:sexMale                     0.8476040 20.4640559
## sesDo not know/do not wish to answer:sexMale 0.1591458  4.9314870
```

#### depression

```
#model: interaction ses*sex
model_interact2 <- glm(hscl_dep_dich ~ ses*sex, family = "binomial", data = data_complete)
summary(model_interact2)
```

```
## 
## Call:
## glm(formula = hscl_dep_dich ~ ses * sex, family = "binomial", 
##     data = data_complete)
## 
## Coefficients:
##                                              Estimate Std. Error z value
## (Intercept)                                   1.52488    0.18141   8.406
## sesBelow average                              0.79189    0.43564   1.818
## sesDo not know/do not wish to answer          0.58533    0.55964   1.046
## sexMale                                      -1.25825    0.28629  -4.395
## sesBelow average:sexMale                     -0.08912    0.60351  -0.148
## sesDo not know/do not wish to answer:sexMale  0.24665    0.89816   0.275
##                                              Pr(>|z|)    
## (Intercept)                                   < 2e-16 ***
## sesBelow average                               0.0691 .  
## sesDo not know/do not wish to answer           0.2956    
## sexMale                                      1.11e-05 ***
## sesBelow average:sexMale                       0.8826    
## sesDo not know/do not wish to answer:sexMale   0.7836    
## ---
## Signif. codes:  0 '***' 0.001 '**' 0.01 '*' 0.05 '.' 0.1 ' ' 1
## 
## (Dispersion parameter for binomial family taken to be 1)
## 
##     Null deviance: 475.08  on 456  degrees of freedom
## Residual deviance: 440.97  on 451  degrees of freedom
## AIC: 452.97
## 
## Number of Fisher Scoring iterations: 4
```

```
exp(coefficients(model_interact2))
```

```
##                                  (Intercept) 
##                                    4.5945946 
##                             sesBelow average 
##                                    2.2075627 
##         sesDo not know/do not wish to answer 
##                                    1.7955882 
##                                      sexMale 
##                                    0.2841503 
##                     sesBelow average:sexMale 
##                                    0.9147384 
## sesDo not know/do not wish to answer:sexMale 
##                                    1.2797323
```

```
exp(confint(model_interact2))
```

```
##                                                  2.5 %   97.5 %
## (Intercept)                                  3.2603873 6.653140
## sesBelow average                             0.9928868 5.614522
## sesDo not know/do not wish to answer         0.6628903 6.289307
## sexMale                                      0.1614369 0.497228
## sesBelow average:sexMale                     0.2710431 2.946872
## sesDo not know/do not wish to answer:sexMale 0.2171382 8.081003
```

### Interaction post-hocs

```
table(data_complete$sex,data_complete$ses)
```

```
##         
##          On or above average Below average Do not know/do not wish to answer
##   Female                 207            78                                37
##   Male                    83            40                                12
```

#### anxiety

```
# we first assess the significance of the interaction with a likelihood ratio test
model_anxiety_main <- glm(hscl_anx_dich ~ ses + sex, family = "binomial", data = data_complete)
model_anxiety_interaction <- glm(hscl_anx_dich ~ ses * sex, family = "binomial", data = data_complete)
epiDisplay::lrtest(model_anxiety_main, model_anxiety_interaction)
```

```
## Likelihood ratio test for MLE method 
## Chi-squared 2 d.f. =  3.156292 , P value =  0.2063573
```

```
# -> check if the interaction term as a whole is statistically significant

plot(effects::allEffects(model_anxiety_main), multiline = TRUE, ci.style = "bars", rescale.axis = FALSE)
```

```
plot(effects::allEffects(model_anxiety_interaction), multiline = TRUE, ci.style = "bars", rescale.axis = FALSE)
```

```
emm_anxiety <- emmeans::emmeans(model_anxiety_interaction, ~ sex | ses)
pairs_anxiety <- summary(pairs(emm_anxiety, ratios = TRUE, type = "response", reverse = TRUE))
pairs_anxiety$LCIL <- exp(log(pairs_anxiety$odds.ratio) - 1.96 * pairs_anxiety$SE)
pairs_anxiety$UCIL <- exp(log(pairs_anxiety$odds.ratio) + 1.96 * pairs_anxiety$SE)

# now we adjust for multiple comparisons with bonferroni correction
pairs_anxiety <- cbind(pairs_anxiety, mutoss::bonferroni(pairs_anxiety$p.value))
pairs_anxiety # final table with results
```

```
##        contrast                               ses odds.ratio         SE  df
## 1 Male / Female               On or above average  0.2705882 0.07733123 Inf
## 2 Male / Female                     Below average  1.0277778 0.75589905 Inf
## 3 Male / Female Do not know/do not wish to answer  0.2424242 0.19624434 Inf
##   null     z.ratio      p.value      LCIL      UCIL   adjPValues
## 1    1 -4.57384840 4.788464e-06 0.2325323 0.3148724 1.436539e-05
## 2    1  0.03725373 9.702827e-01 0.2335958 4.5220305 1.000000e+00
## 3    1 -1.75052774 8.002729e-02 0.1650173 0.3561415 2.400819e-01
```

#### depression

```
# we first assess the significance of the interaction with a likelihood ratio test
model_depression_main <- glm(hscl_dep_dich ~ ses + sex, family = "binomial", data = data_complete)
model_depression_interact <- glm(hscl_dep_dich ~ ses * sex, family = "binomial", data = data_complete)
epiDisplay::lrtest(model_depression_main, model_depression_interact)
```

```
## Likelihood ratio test for MLE method 
## Chi-squared 2 d.f. =  0.1125434 , P value =  0.9452823
```

```
# -> check if the interaction term as a whole is statistically significant

plot(effects::allEffects(model_depression_main), multiline = TRUE, ci.style = "bars", rescale.axis = FALSE)
```

```
plot(effects::allEffects(model_depression_interact), multiline = TRUE, ci.style = "bars", rescale.axis = FALSE)
```

```
emm_depression <- emmeans::emmeans(model_depression_interact, ~ sex | ses)
pairs_depression <- summary(pairs(emm_depression, ratios = TRUE, type = "response", reverse = TRUE))
pairs_depression$LCIL <- exp(log(pairs_depression$odds.ratio) - 1.96 * pairs_depression$SE)
pairs_depression$UCIL <- exp(log(pairs_depression$odds.ratio) + 1.96 * pairs_depression$SE)

# now we adjust for multiple comparisons with bonferroni correction
pairs_depression <- cbind(pairs_depression, mutoss::bonferroni(pairs_depression$p.value))
pairs_depression # final table with results
```

```
##        contrast                               ses odds.ratio         SE  df
## 1 Male / Female               On or above average  0.2841503 0.08135028 Inf
## 2 Male / Female                     Below average  0.2599232 0.13809274 Inf
## 3 Male / Female Do not know/do not wish to answer  0.3636364 0.30956703 Inf
##   null   z.ratio      p.value      LCIL      UCIL   adjPValues
## 1    1 -4.394978 1.107842e-05 0.2422710 0.3332690 3.323525e-05
## 2    1 -2.536067 1.121052e-02 0.1982888 0.3407156 3.363155e-02
## 3    1 -1.188288 2.347198e-01 0.1982250 0.6670772 7.041595e-01
```

## Complete-case data

```
## data set prep
data_complete <- data %>% 
  dplyr::select(sex, ses, hscl_anx_score, hscl_depres_score) %>% 
  dplyr::filter(complete.cases(.))

data_complete <- data_complete %>% 
  dplyr::mutate(hscl_anx_dich = dplyr::case_when(
    hscl_anx_score < 1.75 ~ "no",
    hscl_anx_score >= 1.75 ~ "yes"
  )) %>% 
  dplyr::mutate(hscl_anx_dich = as.factor(hscl_anx_dich))

data_complete <- data_complete %>% 
  dplyr::mutate(hscl_dep_dich = dplyr::case_when(
    hscl_depres_score < 1.75 ~ "no",
    hscl_depres_score >= 1.75 ~ "yes"
  )) %>% 
  dplyr::mutate(hscl_dep_dich = as.factor(hscl_dep_dich))

## prep for anaylsis - setting reference levels
data_complete$ses <- factor(data_complete$ses, ordered = F)
data_complete$ses <- relevel(data_complete$ses, ref = "On or above average")
data_complete$hscl_anx_dich <- relevel(data_complete$hscl_anx_dich, ref = "no")
data_complete$hscl_dep_dich <- relevel(data_complete$hscl_dep_dich, ref = "no")
```

Table 1

```
tab1_complete <- table1::table1(~ ses + hscl_anx_score + hscl_anx_dich + hscl_depres_score + hscl_dep_dich | sex, data = data_complete)
tab1_complete
```

|  | Female (N=310) | Male (N=129) | Overall (N=439) |
| --- | --- | --- | --- |
| ses |  |  |  |
| On or above average | 207 (66.8%) | 83 (64.3%) | 290 (66.1%) |
| Below average | 78 (25.2%) | 40 (31.0%) | 118 (26.9%) |
| Do not know/do not wish to answer | 25 (8.1%) | 6 (4.7%) | 31 (7.1%) |
| hscl\_anx\_score |  |  |  |
| Mean (SD) | 2.54 (0.699) | 2.15 (0.719) | 2.43 (0.727) |
| Median [Min, Max] | 2.50 [1.00, 4.00] | 2.10 [1.00, 3.80] | 2.40 [1.00, 4.00] |
| hscl\_anx\_dich |  |  |  |
| no | 45 (14.5%) | 41 (31.8%) | 86 (19.6%) |
| yes | 265 (85.5%) | 88 (68.2%) | 353 (80.4%) |
| hscl\_depres\_score |  |  |  |
| Mean (SD) | 2.44 (0.648) | 2.04 (0.650) | 2.32 (0.673) |
| Median [Min, Max] | 2.40 [1.00, 4.00] | 2.07 [1.00, 3.67] | 2.33 [1.00, 4.00] |
| hscl\_dep\_dich |  |  |  |
| no | 48 (15.5%) | 49 (38.0%) | 97 (22.1%) |
| yes | 262 (84.5%) | 80 (62.0%) | 342 (77.9%) |

### SES and hscl\_anx

#### Total

```
model_comp1 <- glm(hscl_anx_dich ~ ses, family = "binomial", data = data_complete)
summary(model_comp1)
```

```
## 
## Call:
## glm(formula = hscl_anx_dich ~ ses, family = "binomial", data = data_complete)
## 
## Coefficients:
##                                      Estimate Std. Error z value Pr(>|z|)    
## (Intercept)                            1.0712     0.1347   7.953 1.82e-15 ***
## sesBelow average                       1.4229     0.3721   3.824 0.000131 ***
## sesDo not know/do not wish to answer   1.1624     0.6222   1.868 0.061757 .  
## ---
## Signif. codes:  0 '***' 0.001 '**' 0.01 '*' 0.05 '.' 0.1 ' ' 1
## 
## (Dispersion parameter for binomial family taken to be 1)
## 
##     Null deviance: 434.32  on 438  degrees of freedom
## Residual deviance: 412.74  on 436  degrees of freedom
## AIC: 418.74
## 
## Number of Fisher Scoring iterations: 5
```

```
exp(coefficients(model_comp1))
```

```
##                          (Intercept)                     sesBelow average 
##                             2.918919                             4.149177 
## sesDo not know/do not wish to answer 
##                             3.197531
```

```
exp(confint(model_comp1))
```

```
##                                         2.5 %    97.5 %
## (Intercept)                          2.253542  3.824142
## sesBelow average                     2.100754  9.181383
## sesDo not know/do not wish to answer 1.091660 13.642068
```

```
mod1 <- lm(hscl_anx_score~ses, data = data_complete)
anova(mod1)
```

```
## Analysis of Variance Table
## 
## Response: hscl_anx_score
##            Df  Sum Sq Mean Sq F value   Pr(>F)   
## ses         2   5.626 2.81310  5.4325 0.004673 **
## Residuals 436 225.772 0.51783                    
## ---
## Signif. codes:  0 '***' 0.001 '**' 0.01 '*' 0.05 '.' 0.1 ' ' 1
```

#### Males

```
data_completem <- data_complete %>% 
  dplyr::filter(sex == "Male")

model_comp1m <- glm(hscl_anx_dich ~ ses, family = "binomial", data = data_completem)
summary(model_comp1m)
```

```
## 
## Call:
## glm(formula = hscl_anx_dich ~ ses, family = "binomial", data = data_completem)
## 
## Coefficients:
##                                      Estimate Std. Error z value Pr(>|z|)    
## (Intercept)                            0.2177     0.2208   0.986 0.324168    
## sesBelow average                       2.2946     0.6396   3.587 0.000334 ***
## sesDo not know/do not wish to answer   1.3917     1.1175   1.245 0.212984    
## ---
## Signif. codes:  0 '***' 0.001 '**' 0.01 '*' 0.05 '.' 0.1 ' ' 1
## 
## (Dispersion parameter for binomial family taken to be 1)
## 
##     Null deviance: 161.31  on 128  degrees of freedom
## Residual deviance: 140.80  on 126  degrees of freedom
## AIC: 146.8
## 
## Number of Fisher Scoring iterations: 5
```

```
exp(coefficients(model_comp1m))
```

```
##                          (Intercept)                     sesBelow average 
##                             1.243243                             9.920290 
## sesDo not know/do not wish to answer 
##                             4.021739
```

```
exp(confint(model_comp1m))
```

```
##                                          2.5 %    97.5 %
## (Intercept)                          0.8078365  1.926405
## sesBelow average                     3.2488901 43.374844
## sesDo not know/do not wish to answer 0.6133679 78.848655
```

```
mod1m <- lm(hscl_anx_score~ses, data = data_completem)
anova(mod1m)
```

```
## Analysis of Variance Table
## 
## Response: hscl_anx_score
##            Df Sum Sq Mean Sq F value   Pr(>F)   
## ses         2  5.847 2.92343  6.1011 0.002957 **
## Residuals 126 60.375 0.47917                    
## ---
## Signif. codes:  0 '***' 0.001 '**' 0.01 '*' 0.05 '.' 0.1 ' ' 1
```

#### Females

```
data_completef <- data_complete %>% 
  dplyr::filter(sex == "Female")

model_comp1f <- glm(hscl_anx_dich ~ ses, family = "binomial", data = data_completef)
summary(model_comp1f)
```

```
## 
## Call:
## glm(formula = hscl_anx_dich ~ ses, family = "binomial", data = data_completef)
## 
## Coefficients:
##                                      Estimate Std. Error z value Pr(>|z|)    
## (Intercept)                            1.5249     0.1814   8.406   <2e-16 ***
## sesBelow average                       0.9600     0.4620   2.078   0.0377 *  
## sesDo not know/do not wish to answer   0.9175     0.7592   1.208   0.2269    
## ---
## Signif. codes:  0 '***' 0.001 '**' 0.01 '*' 0.05 '.' 0.1 ' ' 1
## 
## (Dispersion parameter for binomial family taken to be 1)
## 
##     Null deviance: 256.82  on 309  degrees of freedom
## Residual deviance: 250.61  on 307  degrees of freedom
## AIC: 256.61
## 
## Number of Fisher Scoring iterations: 5
```

```
exp(coefficients(model_comp1f))
```

```
##                          (Intercept)                     sesBelow average 
##                             4.594595                             2.611765 
## sesDo not know/do not wish to answer 
##                             2.502941
```

```
exp(confint(model_comp1f))
```

```
##                                          2.5 %    97.5 %
## (Intercept)                          3.2603873  6.653140
## sesBelow average                     1.1293581  7.123744
## sesDo not know/do not wish to answer 0.6977151 16.023354
```

```
mod1f <- lm(hscl_anx_score~ses, data = data_completef)
anova(mod1f)
```

```
## Analysis of Variance Table
## 
## Response: hscl_anx_score
##            Df  Sum Sq Mean Sq F value Pr(>F)
## ses         2   1.765 0.88231  1.8151 0.1646
## Residuals 307 149.233 0.48610
```

### SES and hscl\_depres

#### Total

```
model_comp2 <- glm(hscl_dep_dich ~ ses, family = "binomial", data = data_complete)
summary(model_comp2)
```

```
## 
## Call:
## glm(formula = hscl_dep_dich ~ ses, family = "binomial", data = data_complete)
## 
## Coefficients:
##                                      Estimate Std. Error z value Pr(>|z|)    
## (Intercept)                            1.0894     0.1353   8.052 8.16e-16 ***
## sesBelow average                       0.6254     0.2896   2.159   0.0308 *  
## sesDo not know/do not wish to answer   0.3377     0.4743   0.712   0.4765    
## ---
## Signif. codes:  0 '***' 0.001 '**' 0.01 '*' 0.05 '.' 0.1 ' ' 1
## 
## (Dispersion parameter for binomial family taken to be 1)
## 
##     Null deviance: 463.69  on 438  degrees of freedom
## Residual deviance: 458.50  on 436  degrees of freedom
## AIC: 464.5
## 
## Number of Fisher Scoring iterations: 4
```

```
exp(coefficients(model_comp2))
```

```
##                          (Intercept)                     sesBelow average 
##                             2.972603                             1.868920 
## sesDo not know/do not wish to answer 
##                             1.401690
```

```
exp(confint(model_comp2))
```

```
##                                         2.5 %   97.5 %
## (Intercept)                          2.292551 3.899649
## sesBelow average                     1.079640 3.379070
## sesDo not know/do not wish to answer 0.587390 3.892948
```

```
mod2 <- lm(hscl_depres_score~ses, data = data_complete)
anova(mod2)
```

```
## Analysis of Variance Table
## 
## Response: hscl_depres_score
##            Df  Sum Sq Mean Sq F value  Pr(>F)  
## ses         2   3.481 1.74067  3.8995 0.02096 *
## Residuals 436 194.624 0.44639                  
## ---
## Signif. codes:  0 '***' 0.001 '**' 0.01 '*' 0.05 '.' 0.1 ' ' 1
```

#### Males

```
model_comp2m <- glm(hscl_dep_dich ~ ses, family = "binomial", data = data_completem)
summary(model_comp2m)
```

```
## 
## Call:
## glm(formula = hscl_dep_dich ~ ses, family = "binomial", data = data_completem)
## 
## Coefficients:
##                                      Estimate Std. Error z value Pr(>|z|)  
## (Intercept)                            0.2666     0.2215   1.204   0.2287  
## sesBelow average                       0.7028     0.4177   1.683   0.0925 .
## sesDo not know/do not wish to answer   0.4265     0.8939   0.477   0.6333  
## ---
## Signif. codes:  0 '***' 0.001 '**' 0.01 '*' 0.05 '.' 0.1 ' ' 1
## 
## (Dispersion parameter for binomial family taken to be 1)
## 
##     Null deviance: 171.31  on 128  degrees of freedom
## Residual deviance: 168.29  on 126  degrees of freedom
## AIC: 174.29
## 
## Number of Fisher Scoring iterations: 4
```

```
exp(coefficients(model_comp2m))
```

```
##                          (Intercept)                     sesBelow average 
##                             1.305556                             2.019342 
## sesDo not know/do not wish to answer 
##                             1.531915
```

```
exp(confint(model_comp2m))
```

```
##                                          2.5 %    97.5 %
## (Intercept)                          0.8478901  2.027164
## sesBelow average                     0.9083158  4.722601
## sesDo not know/do not wish to answer 0.2826491 11.491118
```

```
mod2m <- lm(hscl_depres_score~ses, data = data_completem)
anova(mod2m)
```

```
## Analysis of Variance Table
## 
## Response: hscl_depres_score
##            Df Sum Sq Mean Sq F value  Pr(>F)  
## ses         2  1.956 0.97787  2.3643 0.09817 .
## Residuals 126 52.112 0.41359                  
## ---
## Signif. codes:  0 '***' 0.001 '**' 0.01 '*' 0.05 '.' 0.1 ' ' 1
```

#### Females

```
model_comp2f <- glm(hscl_dep_dich ~ ses, family = "binomial", data = data_completef)
summary(model_comp2f)
```

```
## 
## Call:
## glm(formula = hscl_dep_dich ~ ses, family = "binomial", data = data_completef)
## 
## Coefficients:
##                                      Estimate Std. Error z value Pr(>|z|)    
## (Intercept)                            1.5249     0.1814   8.406   <2e-16 ***
## sesBelow average                       0.7919     0.4356   1.818   0.0691 .  
## sesDo not know/do not wish to answer   0.1333     0.5749   0.232   0.8166    
## ---
## Signif. codes:  0 '***' 0.001 '**' 0.01 '*' 0.05 '.' 0.1 ' ' 1
## 
## (Dispersion parameter for binomial family taken to be 1)
## 
##     Null deviance: 267.23  on 309  degrees of freedom
## Residual deviance: 263.45  on 307  degrees of freedom
## AIC: 269.45
## 
## Number of Fisher Scoring iterations: 4
```

```
exp(coefficients(model_comp2f))
```

```
##                          (Intercept)                     sesBelow average 
##                             4.594595                             2.207563 
## sesDo not know/do not wish to answer 
##                             1.142647
```

```
exp(confint(model_comp2f))
```

```
##                                          2.5 %   97.5 %
## (Intercept)                          3.2603873 6.653140
## sesBelow average                     0.9928868 5.614522
## sesDo not know/do not wish to answer 0.4054644 4.088599
```

```
mod2f <- lm(hscl_depres_score~ses, data = data_completef)
anova(mod2f)
```

```
## Analysis of Variance Table
## 
## Response: hscl_depres_score
##            Df  Sum Sq Mean Sq F value  Pr(>F)  
## ses         2   2.161 1.08026  2.6017 0.07578 .
## Residuals 307 127.472 0.41522                  
## ---
## Signif. codes:  0 '***' 0.001 '**' 0.01 '*' 0.05 '.' 0.1 ' ' 1
```

### interaction term ses\*sex

#### anxiety

```
#model: interaction ses*sex
model_interact1 <- glm(hscl_anx_dich ~ ses*sex, family = "binomial", data = data_complete)
summary(model_interact1)
```

```
## 
## Call:
## glm(formula = hscl_anx_dich ~ ses * sex, family = "binomial", 
##     data = data_complete)
## 
## Coefficients:
##                                              Estimate Std. Error z value
## (Intercept)                                    1.5249     0.1814   8.406
## sesBelow average                               0.9600     0.4620   2.078
## sesDo not know/do not wish to answer           0.9175     0.7592   1.208
## sexMale                                       -1.3072     0.2858  -4.574
## sesBelow average:sexMale                       1.3346     0.7890   1.691
## sesDo not know/do not wish to answer:sexMale   0.4742     1.3510   0.351
##                                              Pr(>|z|)    
## (Intercept)                                   < 2e-16 ***
## sesBelow average                               0.0377 *  
## sesDo not know/do not wish to answer           0.2269    
## sexMale                                      4.79e-06 ***
## sesBelow average:sexMale                       0.0908 .  
## sesDo not know/do not wish to answer:sexMale   0.7256    
## ---
## Signif. codes:  0 '***' 0.001 '**' 0.01 '*' 0.05 '.' 0.1 ' ' 1
## 
## (Dispersion parameter for binomial family taken to be 1)
## 
##     Null deviance: 434.32  on 438  degrees of freedom
## Residual deviance: 391.41  on 433  degrees of freedom
## AIC: 403.41
## 
## Number of Fisher Scoring iterations: 5
```

```
exp(coefficients(model_interact1))
```

```
##                                  (Intercept) 
##                                    4.5945946 
##                             sesBelow average 
##                                    2.6117647 
##         sesDo not know/do not wish to answer 
##                                    2.5029412 
##                                      sexMale 
##                                    0.2705882 
##                     sesBelow average:sexMale 
##                                    3.7983092 
## sesDo not know/do not wish to answer:sexMale 
##                                    1.6068053
```

```
exp(confint(model_interact1))
```

```
##                                                  2.5 %     97.5 %
## (Intercept)                                  3.2603873  6.6531404
## sesBelow average                             1.1293581  7.1237438
## sesDo not know/do not wish to answer         0.6977151 16.0233537
## sexMale                                      0.1538128  0.4727804
## sesBelow average:sexMale                     0.8476040 20.4640559
## sesDo not know/do not wish to answer:sexMale 0.1194386 39.9809818
```

#### depression

```
#model: interaction ses*sex
model_interact2 <- glm(hscl_dep_dich ~ ses*sex, family = "binomial", data = data_complete)
summary(model_interact2)
```

```
## 
## Call:
## glm(formula = hscl_dep_dich ~ ses * sex, family = "binomial", 
##     data = data_complete)
## 
## Coefficients:
##                                              Estimate Std. Error z value
## (Intercept)                                   1.52488    0.18141   8.406
## sesBelow average                              0.79189    0.43564   1.818
## sesDo not know/do not wish to answer          0.13335    0.57492   0.232
## sexMale                                      -1.25825    0.28629  -4.395
## sesBelow average:sexMale                     -0.08912    0.60351  -0.148
## sesDo not know/do not wish to answer:sexMale  0.29317    1.06282   0.276
##                                              Pr(>|z|)    
## (Intercept)                                   < 2e-16 ***
## sesBelow average                               0.0691 .  
## sesDo not know/do not wish to answer           0.8166    
## sexMale                                      1.11e-05 ***
## sesBelow average:sexMale                       0.8826    
## sesDo not know/do not wish to answer:sexMale   0.7827    
## ---
## Signif. codes:  0 '***' 0.001 '**' 0.01 '*' 0.05 '.' 0.1 ' ' 1
## 
## (Dispersion parameter for binomial family taken to be 1)
## 
##     Null deviance: 463.69  on 438  degrees of freedom
## Residual deviance: 431.74  on 433  degrees of freedom
## AIC: 443.74
## 
## Number of Fisher Scoring iterations: 4
```

```
exp(coefficients(model_interact2))
```

```
##                                  (Intercept) 
##                                    4.5945946 
##                             sesBelow average 
##                                    2.2075627 
##         sesDo not know/do not wish to answer 
##                                    1.1426471 
##                                      sexMale 
##                                    0.2841503 
##                     sesBelow average:sexMale 
##                                    0.9147384 
## sesDo not know/do not wish to answer:sexMale 
##                                    1.3406720
```

```
exp(confint(model_interact2))
```

```
##                                                  2.5 %    97.5 %
## (Intercept)                                  3.2603873  6.653140
## sesBelow average                             0.9928868  5.614522
## sesDo not know/do not wish to answer         0.4054644  4.088599
## sexMale                                      0.1614369  0.497228
## sesBelow average:sexMale                     0.2710431  2.946872
## sesDo not know/do not wish to answer:sexMale 0.1681415 12.749116
```

### Interaction post-hocs by TG

```
table(data_complete$sex,data_complete$ses) # n = 6 in synthetic dataset
```

```
##         
##          On or above average Below average Do not know/do not wish to answer
##   Female                 207            78                                25
##   Male                    83            40                                 6
```

#### anxiety

```
# we first assess the significance of the interaction with a likelihood ratio test
model_anxiety_main <- glm(hscl_anx_dich ~ ses + sex, family = "binomial", data = data_complete)
model_anxiety_interaction <- glm(hscl_anx_dich ~ ses * sex, family = "binomial", data = data_complete)
epiDisplay::lrtest(model_anxiety_main, model_anxiety_interaction)
```

```
## Likelihood ratio test for MLE method 
## Chi-squared 2 d.f. =  3.08109 , P value =  0.2142643
```

```
# -> check if the interaction term as a whole is statistically significant


plot(effects::allEffects(model_anxiety_main), multiline = TRUE, ci.style = "bars", rescale.axis = FALSE)
```

```
plot(effects::allEffects(model_anxiety_interaction), multiline = TRUE, ci.style = "bars", rescale.axis = FALSE)
```

```
emm_anxiety <- emmeans::emmeans(model_anxiety_interaction, ~ sex | ses)
pairs_anxiety <- summary(pairs(emm_anxiety, ratios = TRUE, type = "response", reverse = TRUE))
pairs_anxiety$LCIL <- exp(log(pairs_anxiety$odds.ratio) - 1.96 * pairs_anxiety$SE)
pairs_anxiety$UCIL <- exp(log(pairs_anxiety$odds.ratio) + 1.96 * pairs_anxiety$SE)

# now we adjust for multiple comparisons with bonferroni correction
pairs_anxiety <- cbind(pairs_anxiety, mutoss::bonferroni(pairs_anxiety$p.value))
pairs_anxiety # final table with results
```

```
##        contrast                               ses odds.ratio         SE  df
## 1 Male / Female               On or above average  0.2705882 0.07733123 Inf
## 2 Male / Female                     Below average  1.0277778 0.75589905 Inf
## 3 Male / Female Do not know/do not wish to answer  0.4347826 0.57409054 Inf
##   null     z.ratio      p.value      LCIL      UCIL   adjPValues
## 1    1 -4.57384840 4.788464e-06 0.2325323 0.3148724 1.436539e-05
## 2    1  0.03725373 9.702827e-01 0.2335958 4.5220305 1.000000e+00
## 3    1 -0.63079667 5.281735e-01 0.1411226 1.3395160 1.000000e+00
```

#### depression

```
# we first assess the significance of the interaction with a likelihood ratio test
model_depression_main <- glm(hscl_dep_dich ~ ses + sex, family = "binomial", data = data_complete)
model_depression_interact <- glm(hscl_dep_dich ~ ses * sex, family = "binomial", data = data_complete)
epiDisplay::lrtest(model_depression_main, model_depression_interact)
```

```
## Likelihood ratio test for MLE method 
## Chi-squared 2 d.f. =  0.1110285 , P value =  0.9459985
```

```
# -> check if the interaction term as a whole is statistically significant

plot(effects::allEffects(model_depression_main), multiline = TRUE, ci.style = "bars", rescale.axis = FALSE)
```

```
plot(effects::allEffects(model_depression_interact), multiline = TRUE, ci.style = "bars", rescale.axis = FALSE)
```

```
emm_depression <- emmeans::emmeans(model_depression_interact, ~ sex | ses)
pairs_depression <- summary(pairs(emm_depression, ratios = TRUE, type = "response", reverse = TRUE))
pairs_depression$LCIL <- exp(log(pairs_depression$odds.ratio) - 1.96 * pairs_depression$SE)
pairs_depression$UCIL <- exp(log(pairs_depression$odds.ratio) + 1.96 * pairs_depression$SE)

# now we adjust for multiple comparisons with bonferroni correction
pairs_depression <- cbind(pairs_depression, mutoss::bonferroni(pairs_depression$p.value))
pairs_depression # final table with results'
```

```
##        contrast                               ses odds.ratio         SE  df
## 1 Male / Female               On or above average  0.2841503 0.08135028 Inf
## 2 Male / Female                     Below average  0.2599232 0.13809274 Inf
## 3 Male / Female Do not know/do not wish to answer  0.3809524 0.38991714 Inf
##   null    z.ratio      p.value      LCIL      UCIL   adjPValues
## 1    1 -4.3949776 1.107842e-05 0.2422710 0.3332690 3.323525e-05
## 2    1 -2.5360673 1.121052e-02 0.1982888 0.3407156 3.363155e-02
## 3    1 -0.9428923 3.457360e-01 0.1774053 0.8180406 1.000000e+00
```

## Sensitivity Analysis: different cut-off for HSCL scores

(reference levels: ses “On average”, hscl < cutoff (depression:
2.1, anxiety: 2.0))

```
data_sens <- data %>% 
  dplyr::select(sex, ses, hscl_anx_score, hscl_depres_score) %>% 
  dplyr::mutate(ses = expss::if_na(ses, "Do not know/do not wish to answer")) %>% 
  dplyr::filter(complete.cases(.))

data_sens <- data_sens %>% 
  dplyr::mutate(hscl_anx_dich = dplyr::case_when(
    hscl_anx_score < 2.0 ~ "no",
    hscl_anx_score >= 2.0 ~ "yes"
  )) %>% 
  dplyr::mutate(hscl_anx_dich = as.factor(hscl_anx_dich))

data_sens <- data_sens %>% 
  dplyr::mutate(hscl_dep_dich = dplyr::case_when(
    hscl_depres_score < 2.1 ~ "no",
    hscl_depres_score >= 2.1 ~ "yes"
  )) %>% 
  dplyr::mutate(hscl_dep_dich = as.factor(hscl_dep_dich))
```

### SES and hscl\_anx

#### Total

```
#setting reference levels for models
data_sens$ses <- factor(data_sens$ses, ordered = F)
data_sens$ses <- relevel(data_sens$ses, ref = "On or above average")
data_sens$hscl_anx_dich <- relevel(data_sens$hscl_anx_dich, ref = "no")
data_sens$hscl_dep_dich <- relevel(data_sens$hscl_dep_dich, ref = "no")

# models: total population - hscl ~ ses
model_sens1 <- glm(hscl_anx_dich ~ ses, family = "binomial", data = data_sens)
summary(model_sens1)
```

```
## 
## Call:
## glm(formula = hscl_anx_dich ~ ses, family = "binomial", data = data_sens)
## 
## Coefficients:
##                                      Estimate Std. Error z value Pr(>|z|)    
## (Intercept)                            0.7824     0.1265   6.183 6.29e-10 ***
## sesBelow average                       0.5313     0.2584   2.056   0.0398 *  
## sesDo not know/do not wish to answer   0.5785     0.3764   1.537   0.1243    
## ---
## Signif. codes:  0 '***' 0.001 '**' 0.01 '*' 0.05 '.' 0.1 ' ' 1
## 
## (Dispersion parameter for binomial family taken to be 1)
## 
##     Null deviance: 538.22  on 456  degrees of freedom
## Residual deviance: 532.28  on 454  degrees of freedom
## AIC: 538.28
## 
## Number of Fisher Scoring iterations: 4
```

ORs and CIs:

```
exp(coefficients(model_sens1))
```

```
##                          (Intercept)                     sesBelow average 
##                             2.186813                             1.701106 
## sesDo not know/do not wish to answer 
##                             1.783417
```

```
exp(confint(model_sens1))
```

```
##                                          2.5 %   97.5 %
## (Intercept)                          1.7124307 2.814083
## sesBelow average                     1.0372238 2.865943
## sesDo not know/do not wish to answer 0.8834027 3.919643
```

#### Males

```
#only males: hscl~ses
data_sensm <- data_sens %>% 
  dplyr::filter(sex == "Male")

model_sens1m <- glm(hscl_anx_dich ~ ses, family = "binomial", data = data_sensm)
summary(model_sens1m)
```

```
## 
## Call:
## glm(formula = hscl_anx_dich ~ ses, family = "binomial", data = data_sensm)
## 
## Coefficients:
##                                      Estimate Std. Error z value Pr(>|z|)  
## (Intercept)                            0.0241     0.2195   0.110   0.9126  
## sesBelow average                       0.8232     0.4090   2.013   0.0441 *
## sesDo not know/do not wish to answer  -0.0241     0.6177  -0.039   0.9689  
## ---
## Signif. codes:  0 '***' 0.001 '**' 0.01 '*' 0.05 '.' 0.1 ' ' 1
## 
## (Dispersion parameter for binomial family taken to be 1)
## 
##     Null deviance: 185.00  on 134  degrees of freedom
## Residual deviance: 180.56  on 132  degrees of freedom
## AIC: 186.56
## 
## Number of Fisher Scoring iterations: 4
```

```
exp(coefficients(model_sens1m))
```

```
##                          (Intercept)                     sesBelow average 
##                            1.0243902                            2.2777778 
## sesDo not know/do not wish to answer 
##                            0.9761905
```

```
exp(confint(model_sens1m))
```

```
##                                          2.5 %   97.5 %
## (Intercept)                          0.6653132 1.578446
## sesBelow average                     1.0395502 5.215444
## sesDo not know/do not wish to answer 0.2836339 3.359501
```

#### Females

```
#only females: hscl~ses
data_sensf <- data_sens %>% 
  dplyr::filter(sex == "Female")

model_sens1f <- glm(hscl_anx_dich ~ ses, family = "binomial", data = data_sensf)
summary(model_sens1f)
```

```
## 
## Call:
## glm(formula = hscl_anx_dich ~ ses, family = "binomial", data = data_sensf)
## 
## Coefficients:
##                                      Estimate Std. Error z value Pr(>|z|)    
## (Intercept)                            1.1442     0.1624   7.046 1.84e-12 ***
## sesBelow average                       0.4652     0.3445   1.350   0.1769    
## sesDo not know/do not wish to answer   0.9660     0.5538   1.744   0.0811 .  
## ---
## Signif. codes:  0 '***' 0.001 '**' 0.01 '*' 0.05 '.' 0.1 ' ' 1
## 
## (Dispersion parameter for binomial family taken to be 1)
## 
##     Null deviance: 329.34  on 321  degrees of freedom
## Residual deviance: 324.52  on 319  degrees of freedom
## AIC: 330.52
## 
## Number of Fisher Scoring iterations: 4
```

```
exp(coefficients(model_sens1f))
```

```
##                          (Intercept)                     sesBelow average 
##                             3.140000                             1.592357 
## sesDo not know/do not wish to answer 
##                             2.627389
```

```
exp(confint(model_sens1f))
```

```
##                                          2.5 %   97.5 %
## (Intercept)                          2.3024556 4.357883
## sesBelow average                     0.8305998 3.235826
## sesDo not know/do not wish to answer 0.9845551 9.126787
```

### SES and hscl\_depres

#### Total

```
model_sens2 <- glm(hscl_dep_dich ~ ses, family = "binomial", data = data_sens)
summary(model_sens2)
```

```
## 
## Call:
## glm(formula = hscl_dep_dich ~ ses, family = "binomial", data = data_sens)
## 
## Coefficients:
##                                      Estimate Std. Error z value Pr(>|z|)   
## (Intercept)                            0.3199     0.1189   2.690  0.00715 **
## sesBelow average                       0.7120     0.2406   2.959  0.00309 **
## sesDo not know/do not wish to answer   0.2237     0.3193   0.700  0.48363   
## ---
## Signif. codes:  0 '***' 0.001 '**' 0.01 '*' 0.05 '.' 0.1 ' ' 1
## 
## (Dispersion parameter for binomial family taken to be 1)
## 
##     Null deviance: 604.28  on 456  degrees of freedom
## Residual deviance: 595.04  on 454  degrees of freedom
## AIC: 601.04
## 
## Number of Fisher Scoring iterations: 4
```

ORs and CIs:

```
exp(coefficients(model_sens2))
```

```
##                          (Intercept)                     sesBelow average 
##                             1.377049                             2.038018 
## sesDo not know/do not wish to answer 
##                             1.250661
```

```
exp(confint(model_sens2))
```

```
##                                          2.5 %   97.5 %
## (Intercept)                          1.0919650 1.741574
## sesBelow average                     1.2826259 3.302227
## sesDo not know/do not wish to answer 0.6751389 2.376969
```

#### Males

```
#only males: hscl~ses
model_sens2m <- glm(hscl_dep_dich ~ ses, family = "binomial", data = data_sensm)
summary(model_sens2m)
```

```
## 
## Call:
## glm(formula = hscl_dep_dich ~ ses, family = "binomial", data = data_sensm)
## 
## Coefficients:
##                                      Estimate Std. Error z value Pr(>|z|)
## (Intercept)                           -0.3159     0.2223  -1.421    0.155
## sesBelow average                       0.6181     0.3895   1.587    0.113
## sesDo not know/do not wish to answer   0.3159     0.6187   0.511    0.610
## 
## (Dispersion parameter for binomial family taken to be 1)
## 
##     Null deviance: 186.79  on 134  degrees of freedom
## Residual deviance: 184.20  on 132  degrees of freedom
## AIC: 190.2
## 
## Number of Fisher Scoring iterations: 4
```

```
exp(coefficients(model_sens2m))
```

```
##                          (Intercept)                     sesBelow average 
##                            0.7291667                            1.8554622 
## sesDo not know/do not wish to answer 
##                            1.3714286
```

```
exp(confint(model_sens2m))
```

```
##                                          2.5 %   97.5 %
## (Intercept)                          0.4684954 1.123617
## sesBelow average                     0.8692096 4.027272
## sesDo not know/do not wish to answer 0.3979942 4.731102
```

#### Females

```
#only females: hscl~ses
model_sens2f <- glm(hscl_dep_dich ~ ses, family = "binomial", data = data_sensf)
summary(model_sens2f)
```

```
## 
## Call:
## glm(formula = hscl_dep_dich ~ ses, family = "binomial", data = data_sensf)
## 
## Coefficients:
##                                      Estimate Std. Error z value Pr(>|z|)    
## (Intercept)                            0.5863     0.1450   4.043 5.29e-05 ***
## sesBelow average                       0.9335     0.3288   2.840  0.00452 ** 
## sesDo not know/do not wish to answer   0.1477     0.3800   0.389  0.69750    
## ---
## Signif. codes:  0 '***' 0.001 '**' 0.01 '*' 0.05 '.' 0.1 ' ' 1
## 
## (Dispersion parameter for binomial family taken to be 1)
## 
##     Null deviance: 398.99  on 321  degrees of freedom
## Residual deviance: 389.95  on 319  degrees of freedom
## AIC: 395.95
## 
## Number of Fisher Scoring iterations: 4
```

```
exp(coefficients(model_sens2f))
```

```
##                          (Intercept)                     sesBelow average 
##                             1.797297                             2.543502 
## sesDo not know/do not wish to answer 
##                             1.159148
```

```
exp(confint(model_sens2f))
```

```
##                                          2.5 %   97.5 %
## (Intercept)                          1.3571649 2.398572
## sesBelow average                     1.3681138 5.004953
## sesDo not know/do not wish to answer 0.5602175 2.514192
```

### interaction term ses\*sex

#### anxiety

```
#model: interaction ses*sex
model_sens_interact1 <- glm(hscl_anx_dich ~ ses*sex, family = "binomial", data = data_sens)
summary(model_sens_interact1)
```

```
## 
## Call:
## glm(formula = hscl_anx_dich ~ ses * sex, family = "binomial", 
##     data = data_sens)
## 
## Coefficients:
##                                              Estimate Std. Error z value
## (Intercept)                                    1.1442     0.1624   7.046
## sesBelow average                               0.4652     0.3445   1.350
## sesDo not know/do not wish to answer           0.9660     0.5538   1.744
## sexMale                                       -1.1201     0.2731  -4.102
## sesBelow average:sexMale                       0.3580     0.5347   0.669
## sesDo not know/do not wish to answer:sexMale  -0.9901     0.8296  -1.193
##                                              Pr(>|z|)    
## (Intercept)                                  1.84e-12 ***
## sesBelow average                               0.1769    
## sesDo not know/do not wish to answer           0.0811 .  
## sexMale                                      4.10e-05 ***
## sesBelow average:sexMale                       0.5032    
## sesDo not know/do not wish to answer:sexMale   0.2327    
## ---
## Signif. codes:  0 '***' 0.001 '**' 0.01 '*' 0.05 '.' 0.1 ' ' 1
## 
## (Dispersion parameter for binomial family taken to be 1)
## 
##     Null deviance: 538.22  on 456  degrees of freedom
## Residual deviance: 505.07  on 451  degrees of freedom
## AIC: 517.07
## 
## Number of Fisher Scoring iterations: 4
```

```
exp(coefficients(model_sens_interact1))
```

```
##                                  (Intercept) 
##                                    3.1400000 
##                             sesBelow average 
##                                    1.5923567 
##         sesDo not know/do not wish to answer 
##                                    2.6273885 
##                                      sexMale 
##                                    0.3262389 
##                     sesBelow average:sexMale 
##                                    1.4304444 
## sesDo not know/do not wish to answer:sexMale 
##                                    0.3715440
```

```
exp(confint(model_sens_interact1))
```

```
##                                                   2.5 %    97.5 %
## (Intercept)                                  2.30245556 4.3578830
## sesBelow average                             0.83059977 3.2358261
## sesDo not know/do not wish to answer         0.98455514 9.1267872
## sexMale                                      0.19027860 0.5561887
## sesBelow average:sexMale                     0.49961766 4.1020919
## sesDo not know/do not wish to answer:sexMale 0.06734607 1.8257666
```

#### depression

```
#model: interaction ses*sex
model_sens_interact2 <- glm(hscl_dep_dich ~ ses*sex, family = "binomial", data = data_sens)
summary(model_sens_interact2)
```

```
## 
## Call:
## glm(formula = hscl_dep_dich ~ ses * sex, family = "binomial", 
##     data = data_sens)
## 
## Coefficients:
##                                              Estimate Std. Error z value
## (Intercept)                                    0.5863     0.1450   4.043
## sesBelow average                               0.9335     0.3288   2.840
## sesDo not know/do not wish to answer           0.1477     0.3800   0.389
## sexMale                                       -0.9021     0.2654  -3.399
## sesBelow average:sexMale                      -0.3154     0.5097  -0.619
## sesDo not know/do not wish to answer:sexMale   0.1682     0.7260   0.232
##                                              Pr(>|z|)    
## (Intercept)                                  5.29e-05 ***
## sesBelow average                             0.004518 ** 
## sesDo not know/do not wish to answer         0.697504    
## sexMale                                      0.000676 ***
## sesBelow average:sexMale                     0.536040    
## sesDo not know/do not wish to answer:sexMale 0.816825    
## ---
## Signif. codes:  0 '***' 0.001 '**' 0.01 '*' 0.05 '.' 0.1 ' ' 1
## 
## (Dispersion parameter for binomial family taken to be 1)
## 
##     Null deviance: 604.28  on 456  degrees of freedom
## Residual deviance: 574.16  on 451  degrees of freedom
## AIC: 586.16
## 
## Number of Fisher Scoring iterations: 4
```

```
exp(coefficients(model_sens_interact2))
```

```
##                                  (Intercept) 
##                                    1.7972973 
##                             sesBelow average 
##                                    2.5435016 
##         sesDo not know/do not wish to answer 
##                                    1.1591479 
##                                      sexMale 
##                                    0.4057018 
##                     sesBelow average:sexMale 
##                                    0.7294913 
## sesDo not know/do not wish to answer:sexMale 
##                                    1.1831351
```

```
exp(confint(model_sens_interact2))
```

```
##                                                  2.5 %    97.5 %
## (Intercept)                                  1.3571649 2.3985718
## sesBelow average                             1.3681138 5.0049531
## sesDo not know/do not wish to answer         0.5602175 2.5141919
## sexMale                                      0.2397725 0.6801127
## sesBelow average:sexMale                     0.2659288 1.9736374
## sesDo not know/do not wish to answer:sexMale 0.2788358 4.9644293
```

## Sensitivity Analysis 2: Extreme cases

### all HSCL NAs “yes”

```
data_yes <- data %>% 
  dplyr::select(sex, ses, hscl_anx_dich, hscl_dep_dich) %>%
  dplyr::mutate(ses = expss::if_na(ses, "Do not know/do not wish to answer")) %>% 
  dplyr::mutate(hscl_anx_dich = expss::if_na(hscl_anx_dich, "yes")) %>% 
  dplyr::mutate(hscl_dep_dich = expss::if_na(hscl_dep_dich, "yes")) %>% 
  dplyr::filter(complete.cases(.))

data_yes$ses <- factor(data_yes$ses, ordered = F)
data_yes$ses <- relevel(data_yes$ses, ref = "On or above average")
data_yes$hscl_anx_dich <- relevel(data_yes$hscl_anx_dich, ref = "no")
data_yes$hscl_dep_dich <- relevel(data_yes$hscl_dep_dich, ref = "no")
```

#### SES and hscl\_anx

##### Total

```
# models: total population - hscl ~ ses
model_yes1 <- glm(hscl_anx_dich ~ ses, family = "binomial", data = data_yes)
summary(model_yes1)
```

```
## 
## Call:
## glm(formula = hscl_anx_dich ~ ses, family = "binomial", data = data_yes)
## 
## Coefficients:
##                                      Estimate Std. Error z value Pr(>|z|)    
## (Intercept)                            1.1399     0.1232   9.255  < 2e-16 ***
## sesBelow average                       1.3697     0.3511   3.901 9.59e-05 ***
## sesDo not know/do not wish to answer   0.4183     0.3407   1.228     0.22    
## ---
## Signif. codes:  0 '***' 0.001 '**' 0.01 '*' 0.05 '.' 0.1 ' ' 1
## 
## (Dispersion parameter for binomial family taken to be 1)
## 
##     Null deviance: 552.46  on 560  degrees of freedom
## Residual deviance: 532.35  on 558  degrees of freedom
## AIC: 538.35
## 
## Number of Fisher Scoring iterations: 5
```

ORs and CIs:

```
exp(coefficients(model_yes1))
```

```
##                          (Intercept)                     sesBelow average 
##                             3.126437                             3.934191 
## sesDo not know/do not wish to answer 
##                             1.519301
```

```
exp(confint(model_yes1))
```

```
##                                          2.5 %   97.5 %
## (Intercept)                          2.4674510 4.001387
## sesBelow average                     2.0670604 8.302993
## sesDo not know/do not wish to answer 0.8037675 3.088969
```

##### Males

```
data_yesm <- data_yes %>% 
  dplyr::filter(sex == "Male")

#only males: hscl~ses
model_yes1m <- glm(hscl_anx_dich ~ ses, family = "binomial", data = data_yesm)
summary(model_yes1m)
```

```
## 
## Call:
## glm(formula = hscl_anx_dich ~ ses, family = "binomial", data = data_yesm)
## 
## Coefficients:
##                                      Estimate Std. Error z value Pr(>|z|)    
## (Intercept)                           0.33647    0.20702   1.625 0.104095    
## sesBelow average                      2.25379    0.63339   3.558 0.000373 ***
## sesDo not know/do not wish to answer -0.04879    0.57838  -0.084 0.932773    
## ---
## Signif. codes:  0 '***' 0.001 '**' 0.01 '*' 0.05 '.' 0.1 ' ' 1
## 
## (Dispersion parameter for binomial family taken to be 1)
## 
##     Null deviance: 191.88  on 152  degrees of freedom
## Residual deviance: 171.29  on 150  degrees of freedom
## AIC: 177.29
## 
## Number of Fisher Scoring iterations: 5
```

```
exp(coefficients(model_yes1m))
```

```
##                          (Intercept)                     sesBelow average 
##                             1.400000                             9.523810 
## sesDo not know/do not wish to answer 
##                             0.952381
```

```
exp(confint(model_yes1m))
```

```
##                                          2.5 %    97.5 %
## (Intercept)                          0.9360445  2.113265
## sesBelow average                     3.1675760 41.281716
## sesDo not know/do not wish to answer 0.3073853  3.093614
```

##### Females

```
data_yesf <- data_yes %>% 
  dplyr::filter(sex == "Female")

#only females: hscl~ses
model_yes1f <- glm(hscl_anx_dich ~ ses, family = "binomial", data = data_yesf)
summary(model_yes1f)
```

```
## 
## Call:
## glm(formula = hscl_anx_dich ~ ses, family = "binomial", data = data_yesf)
## 
## Coefficients:
##                                      Estimate Std. Error z value Pr(>|z|)    
## (Intercept)                            1.5251     0.1610   9.476   <2e-16 ***
## sesBelow average                       0.9478     0.4252   2.229   0.0258 *  
## sesDo not know/do not wish to answer   0.5749     0.4615   1.246   0.2128    
## ---
## Signif. codes:  0 '***' 0.001 '**' 0.01 '*' 0.05 '.' 0.1 ' ' 1
## 
## (Dispersion parameter for binomial family taken to be 1)
## 
##     Null deviance: 340.74  on 407  degrees of freedom
## Residual deviance: 334.02  on 405  degrees of freedom
## AIC: 340.02
## 
## Number of Fisher Scoring iterations: 5
```

```
exp(coefficients(model_yes1f))
```

```
##                          (Intercept)                     sesBelow average 
##                             4.595745                             2.580026 
## sesDo not know/do not wish to answer 
##                             1.777006
```

```
exp(confint(model_yes1f))
```

```
##                                          2.5 %   97.5 %
## (Intercept)                          3.3858826 6.372759
## sesBelow average                     1.1898901 6.455714
## sesDo not know/do not wish to answer 0.7694782 4.843186
```

#### SES and hscl\_depres

##### Total

```
# models: total population - hscl ~ ses
model_yes2 <- glm(hscl_dep_dich ~ ses, family = "binomial", data = data_yes)
summary(model_yes2)
```

```
## 
## Call:
## glm(formula = hscl_dep_dich ~ ses, family = "binomial", data = data_yes)
## 
## Coefficients:
##                                      Estimate Std. Error z value Pr(>|z|)    
## (Intercept)                            1.3655     0.1311  10.414   <2e-16 ***
## sesBelow average                       0.4890     0.2854   1.714   0.0866 .  
## sesDo not know/do not wish to answer   0.8157     0.4197   1.943   0.0520 .  
## ---
## Signif. codes:  0 '***' 0.001 '**' 0.01 '*' 0.05 '.' 0.1 ' ' 1
## 
## (Dispersion parameter for binomial family taken to be 1)
## 
##     Null deviance: 519.76  on 560  degrees of freedom
## Residual deviance: 513.34  on 558  degrees of freedom
## AIC: 519.34
## 
## Number of Fisher Scoring iterations: 4
```

ORs and CIs:

```
exp(coefficients(model_yes2))
```

```
##                          (Intercept)                     sesBelow average 
##                             3.917808                             1.630730 
## sesDo not know/do not wish to answer 
##                             2.260739
```

```
exp(confint(model_yes2))
```

```
##                                          2.5 %   97.5 %
## (Intercept)                          3.0485813 5.101269
## sesBelow average                     0.9505058 2.926577
## sesDo not know/do not wish to answer 1.0564534 5.609055
```

##### Males

```
#only males: hscl~ses
model_yes2m <- glm(hscl_dep_dich ~ ses, family = "binomial", data = data_yesm)
summary(model_yes2m)
```

```
## 
## Call:
## glm(formula = hscl_dep_dich ~ ses, family = "binomial", data = data_yesm)
## 
## Coefficients:
##                                      Estimate Std. Error z value Pr(>|z|)  
## (Intercept)                            0.5108     0.2108   2.423   0.0154 *
## sesBelow average                       0.5570     0.4082   1.365   0.1724  
## sesDo not know/do not wish to answer   0.7885     0.6846   1.152   0.2494  
## ---
## Signif. codes:  0 '***' 0.001 '**' 0.01 '*' 0.05 '.' 0.1 ' ' 1
## 
## (Dispersion parameter for binomial family taken to be 1)
## 
##     Null deviance: 193.36  on 152  degrees of freedom
## Residual deviance: 190.47  on 150  degrees of freedom
## AIC: 196.47
## 
## Number of Fisher Scoring iterations: 4
```

```
exp(coefficients(model_yes2m))
```

```
##                          (Intercept)                     sesBelow average 
##                             1.666667                             1.745455 
## sesDo not know/do not wish to answer 
##                             2.200000
```

```
exp(confint(model_yes2m))
```

```
##                                          2.5 %    97.5 %
## (Intercept)                          1.1087967  2.541320
## sesBelow average                     0.8008180  4.012794
## sesDo not know/do not wish to answer 0.6363736 10.203650
```

##### Females

```
#only females: hscl~ses
model_yes2f <- glm(hscl_dep_dich ~ ses, family = "binomial", data = data_yesf)
summary(model_yes2f)
```

```
## 
## Call:
## glm(formula = hscl_dep_dich ~ ses, family = "binomial", data = data_yesf)
## 
## Coefficients:
##                                      Estimate Std. Error z value Pr(>|z|)    
## (Intercept)                            1.8096     0.1773  10.204   <2e-16 ***
## sesBelow average                       0.6633     0.4317   1.537    0.124    
## sesDo not know/do not wish to answer   0.7359     0.5487   1.341    0.180    
## ---
## Signif. codes:  0 '***' 0.001 '**' 0.01 '*' 0.05 '.' 0.1 ' ' 1
## 
## (Dispersion parameter for binomial family taken to be 1)
## 
##     Null deviance: 295.56  on 407  degrees of freedom
## Residual deviance: 291.53  on 405  degrees of freedom
## AIC: 297.53
## 
## Number of Fisher Scoring iterations: 5
```

```
exp(coefficients(model_yes2f))
```

```
##                          (Intercept)                     sesBelow average 
##                             6.108108                             1.941214 
## sesDo not know/do not wish to answer 
##                             2.087389
```

```
exp(confint(model_yes2f))
```

```
##                                          2.5 %   97.5 %
## (Intercept)                          4.3732964 8.782428
## sesBelow average                     0.8811174 4.905307
## sesDo not know/do not wish to answer 0.7922261 7.197936
```

#### interaction term ses\*sex

##### anxiety

```
#model: interaction ses*sex
model_yes_interact1 <- glm(hscl_anx_dich ~ ses*sex, family = "binomial", data = data_yes)
summary(model_yes_interact1)
```

```
## 
## Call:
## glm(formula = hscl_anx_dich ~ ses * sex, family = "binomial", 
##     data = data_yes)
## 
## Coefficients:
##                                              Estimate Std. Error z value
## (Intercept)                                    1.5251     0.1610   9.476
## sesBelow average                               0.9478     0.4252   2.229
## sesDo not know/do not wish to answer           0.5749     0.4615   1.246
## sexMale                                       -1.1887     0.2622  -4.533
## sesBelow average:sexMale                       1.3060     0.7629   1.712
## sesDo not know/do not wish to answer:sexMale  -0.6237     0.7399  -0.843
##                                              Pr(>|z|)    
## (Intercept)                                   < 2e-16 ***
## sesBelow average                               0.0258 *  
## sesDo not know/do not wish to answer           0.2128    
## sexMale                                      5.82e-06 ***
## sesBelow average:sexMale                       0.0869 .  
## sesDo not know/do not wish to answer:sexMale   0.3993    
## ---
## Signif. codes:  0 '***' 0.001 '**' 0.01 '*' 0.05 '.' 0.1 ' ' 1
## 
## (Dispersion parameter for binomial family taken to be 1)
## 
##     Null deviance: 552.46  on 560  degrees of freedom
## Residual deviance: 505.31  on 555  degrees of freedom
## AIC: 517.31
## 
## Number of Fisher Scoring iterations: 5
```

```
exp(coefficients(model_yes_interact1))
```

```
##                                  (Intercept) 
##                                    4.5957447 
##                             sesBelow average 
##                                    2.5800265 
##         sesDo not know/do not wish to answer 
##                                    1.7770062 
##                                      sexMale 
##                                    0.3046296 
##                     sesBelow average:sexMale 
##                                    3.6913612 
## sesDo not know/do not wish to answer:sexMale 
##                                    0.5359469
```

```
exp(confint(model_yes_interact1))
```

```
##                                                  2.5 %     97.5 %
## (Intercept)                                  3.3858826  6.3727590
## sesBelow average                             1.1898901  6.4557136
## sesDo not know/do not wish to answer         0.7694782  4.8431859
## sexMale                                      0.1818020  0.5092799
## sesBelow average:sexMale                     0.8802025 19.1515597
## sesDo not know/do not wish to answer:sexMale 0.1214364  2.2864189
```

##### depression

```
#model: interaction ses*sex
model_yes_interact2 <- glm(hscl_dep_dich ~ ses*sex, family = "binomial", data = data_yes)
summary(model_yes_interact2)
```

```
## 
## Call:
## glm(formula = hscl_dep_dich ~ ses * sex, family = "binomial", 
##     data = data_yes)
## 
## Coefficients:
##                                              Estimate Std. Error z value
## (Intercept)                                   1.80962    0.17735  10.204
## sesBelow average                              0.66331    0.43169   1.537
## sesDo not know/do not wish to answer          0.73591    0.54869   1.341
## sexMale                                      -1.29879    0.27549  -4.714
## sesBelow average:sexMale                     -0.10630    0.59410  -0.179
## sesDo not know/do not wish to answer:sexMale  0.05254    0.87735   0.060
##                                              Pr(>|z|)    
## (Intercept)                                   < 2e-16 ***
## sesBelow average                                0.124    
## sesDo not know/do not wish to answer            0.180    
## sexMale                                      2.42e-06 ***
## sesBelow average:sexMale                        0.858    
## sesDo not know/do not wish to answer:sexMale    0.952    
## ---
## Signif. codes:  0 '***' 0.001 '**' 0.01 '*' 0.05 '.' 0.1 ' ' 1
## 
## (Dispersion parameter for binomial family taken to be 1)
## 
##     Null deviance: 519.76  on 560  degrees of freedom
## Residual deviance: 482.00  on 555  degrees of freedom
## AIC: 494
## 
## Number of Fisher Scoring iterations: 5
```

```
exp(coefficients(model_yes_interact2))
```

```
##                                  (Intercept) 
##                                    6.1081081 
##                             sesBelow average 
##                                    1.9412137 
##         sesDo not know/do not wish to answer 
##                                    2.0873894 
##                                      sexMale 
##                                    0.2728614 
##                     sesBelow average:sexMale 
##                                    0.8991563 
## sesDo not know/do not wish to answer:sexMale 
##                                    1.0539481
```

```
exp(confint(model_yes_interact2))
```

```
##                                                  2.5 %   97.5 %
## (Intercept)                                  4.3732964 8.782428
## sesBelow average                             0.8811174 4.905307
## sesDo not know/do not wish to answer         0.7922261 7.197936
## sexMale                                      0.1585111 0.468009
## sesBelow average:sexMale                     0.2711045 2.842491
## sesDo not know/do not wish to answer:sexMale 0.1865755 6.432098
```

### all HSCL NAs “no”

```
data_no <- data %>% 
  dplyr::select(sex, ses, hscl_anx_dich, hscl_dep_dich) %>%
  dplyr::mutate(ses = expss::if_na(ses, "Do not know/do not wish to answer")) %>% 
  dplyr::mutate(hscl_anx_dich = expss::if_na(hscl_anx_dich, "no")) %>% 
  dplyr::mutate(hscl_dep_dich = expss::if_na(hscl_dep_dich, "no")) %>% 
  dplyr::filter(complete.cases(.))

data_no$ses <- factor(data_no$ses, ordered = F)
data_no$ses <- relevel(data_no$ses, ref = "On or above average")
data_no$hscl_anx_dich <- relevel(data_no$hscl_anx_dich, ref = "no")
data_no$hscl_dep_dich <- relevel(data_no$hscl_dep_dich, ref = "no")
```

#### SES and hscl\_anx

##### Total

```
# models: total population - hscl ~ ses
model_no1 <- glm(hscl_anx_dich ~ ses, family = "binomial", data = data_no)
summary(model_no1)
```

```
## 
## Call:
## glm(formula = hscl_anx_dich ~ ses, family = "binomial", data = data_no)
## 
## Coefficients:
##                                      Estimate Std. Error z value Pr(>|z|)    
## (Intercept)                            0.8566     0.1154   7.424 1.14e-13 ***
## sesBelow average                       1.2835     0.3052   4.206 2.60e-05 ***
## sesDo not know/do not wish to answer   0.1110     0.2932   0.379    0.705    
## ---
## Signif. codes:  0 '***' 0.001 '**' 0.01 '*' 0.05 '.' 0.1 ' ' 1
## 
## (Dispersion parameter for binomial family taken to be 1)
## 
##     Null deviance: 630.39  on 560  degrees of freedom
## Residual deviance: 608.13  on 558  degrees of freedom
## AIC: 614.13
## 
## Number of Fisher Scoring iterations: 4
```

ORs and CIs:

```
exp(coefficients(model_no1))
```

```
##                          (Intercept)                     sesBelow average 
##                             2.355140                             3.609127 
## sesDo not know/do not wish to answer 
##                             1.117377
```

```
exp(confint(model_no1))
```

```
##                                          2.5 %   97.5 %
## (Intercept)                          1.8844657 2.963767
## sesBelow average                     2.0452534 6.823095
## sesDo not know/do not wish to answer 0.6381812 2.025352
```

##### Males

```
#only females: hscl~ses
data_nom <- data_no %>% 
  dplyr::filter(sex == "Male")

model_no1m <- glm(hscl_anx_dich ~ ses, family = "binomial", data = data_nom)
summary(model_no1m)
```

```
## 
## Call:
## glm(formula = hscl_anx_dich ~ ses, family = "binomial", data = data_nom)
## 
## Coefficients:
##                                      Estimate Std. Error z value Pr(>|z|)    
## (Intercept)                           0.20909    0.20524   1.019 0.308315    
## sesBelow average                      1.61007    0.48561   3.316 0.000915 ***
## sesDo not know/do not wish to answer  0.07859    0.57775   0.136 0.891798    
## ---
## Signif. codes:  0 '***' 0.001 '**' 0.01 '*' 0.05 '.' 0.1 ' ' 1
## 
## (Dispersion parameter for binomial family taken to be 1)
## 
##     Null deviance: 199.85  on 152  degrees of freedom
## Residual deviance: 185.92  on 150  degrees of freedom
## AIC: 191.92
## 
## Number of Fisher Scoring iterations: 4
```

```
exp(coefficients(model_no1m))
```

```
##                          (Intercept)                     sesBelow average 
##                             1.232558                             5.003145 
## sesDo not know/do not wish to answer 
##                             1.081761
```

```
exp(confint(model_no1m))
```

```
##                                          2.5 %    97.5 %
## (Intercept)                          0.8255411  1.850674
## sesBelow average                     2.0515614 14.173126
## sesDo not know/do not wish to answer 0.3496543  3.510561
```

##### Females

```
#only females: hscl~ses
data_nof <- data_no %>% 
  dplyr::filter(sex == "Female")

model_no1f <- glm(hscl_anx_dich ~ ses, family = "binomial", data = data_nof)
summary(model_no1f)
```

```
## 
## Call:
## glm(formula = hscl_anx_dich ~ ses, family = "binomial", data = data_nof)
## 
## Coefficients:
##                                      Estimate Std. Error z value Pr(>|z|)    
## (Intercept)                            1.1344     0.1437   7.894 2.92e-15 ***
## sesBelow average                       1.1929     0.3972   3.003  0.00267 ** 
## sesDo not know/do not wish to answer   0.0383     0.3484   0.110  0.91247    
## ---
## Signif. codes:  0 '***' 0.001 '**' 0.01 '*' 0.05 '.' 0.1 ' ' 1
## 
## (Dispersion parameter for binomial family taken to be 1)
## 
##     Null deviance: 417.58  on 407  degrees of freedom
## Residual deviance: 406.03  on 405  degrees of freedom
## AIC: 412.03
## 
## Number of Fisher Scoring iterations: 4
```

```
exp(coefficients(model_no1f))
```

```
##                          (Intercept)                     sesBelow average 
##                             3.109375                             3.296482 
## sesDo not know/do not wish to answer 
##                             1.039041
```

```
exp(confint(model_no1f))
```

```
##                                          2.5 %   97.5 %
## (Intercept)                          2.3609703 4.151159
## sesBelow average                     1.5956930 7.724134
## sesDo not know/do not wish to answer 0.5368918 2.124457
```

#### SES and hscl\_depres

##### Total

```
# models: total population - hscl ~ ses
model_no2 <- glm(hscl_dep_dich ~ ses, family = "binomial", data = data_no)
summary(model_no2)
```

```
## 
## Call:
## glm(formula = hscl_dep_dich ~ ses, family = "binomial", data = data_no)
## 
## Coefficients:
##                                      Estimate Std. Error z value Pr(>|z|)    
## (Intercept)                           0.51826    0.10912   4.749 2.04e-06 ***
## sesBelow average                      0.59040    0.22850   2.584  0.00977 ** 
## sesDo not know/do not wish to answer -0.01516    0.27134  -0.056  0.95545    
## ---
## Signif. codes:  0 '***' 0.001 '**' 0.01 '*' 0.05 '.' 0.1 ' ' 1
## 
## (Dispersion parameter for binomial family taken to be 1)
## 
##     Null deviance: 722.20  on 560  degrees of freedom
## Residual deviance: 714.81  on 558  degrees of freedom
## AIC: 720.81
## 
## Number of Fisher Scoring iterations: 4
```

ORs and CIs:

```
exp(coefficients(model_no2))
```

```
##                          (Intercept)                     sesBelow average 
##                            1.6791045                            1.8047138 
## sesDo not know/do not wish to answer 
##                            0.9849573
```

```
exp(confint(model_no2))
```

```
##                                          2.5 %   97.5 %
## (Intercept)                          1.3581429 2.083977
## sesBelow average                     1.1631014 2.854941
## sesDo not know/do not wish to answer 0.5821571 1.693123
```

##### Males

```
model_no2m <- glm(hscl_dep_dich ~ ses, family = "binomial", data = data_nom)
summary(model_no2m)
```

```
## 
## Call:
## glm(formula = hscl_dep_dich ~ ses, family = "binomial", data = data_nom)
## 
## Coefficients:
##                                      Estimate Std. Error z value Pr(>|z|)  
## (Intercept)                          -0.04167    0.20417  -0.204   0.8383  
## sesBelow average                      0.76991    0.38418   2.004   0.0451 *
## sesDo not know/do not wish to answer  0.62946    0.59397   1.060   0.2893  
## ---
## Signif. codes:  0 '***' 0.001 '**' 0.01 '*' 0.05 '.' 0.1 ' ' 1
## 
## (Dispersion parameter for binomial family taken to be 1)
## 
##     Null deviance: 210.21  on 152  degrees of freedom
## Residual deviance: 205.56  on 150  degrees of freedom
## AIC: 211.56
## 
## Number of Fisher Scoring iterations: 4
```

```
exp(coefficients(model_no2m))
```

```
##                          (Intercept)                     sesBelow average 
##                            0.9591837                            2.1595745 
## sesDo not know/do not wish to answer 
##                            1.8765957
```

```
exp(confint(model_no2m))
```

```
##                                          2.5 %   97.5 %
## (Intercept)                          0.6416338 1.432289
## sesBelow average                     1.0308648 4.684522
## sesDo not know/do not wish to answer 0.6025412 6.489378
```

##### Females

```
model_no2f <- glm(hscl_dep_dich ~ ses, family = "binomial", data = data_nof)
summary(model_no2f)
```

```
## 
## Call:
## glm(formula = hscl_dep_dich ~ ses, family = "binomial", data = data_nof)
## 
## Coefficients:
##                                      Estimate Std. Error z value Pr(>|z|)    
## (Intercept)                            0.7391     0.1318   5.606 2.07e-08 ***
## sesBelow average                       0.5791     0.2900   1.997   0.0458 *  
## sesDo not know/do not wish to answer  -0.2573     0.3073  -0.837   0.4024    
## ---
## Signif. codes:  0 '***' 0.001 '**' 0.01 '*' 0.05 '.' 0.1 ' ' 1
## 
## (Dispersion parameter for binomial family taken to be 1)
## 
##     Null deviance: 502.79  on 407  degrees of freedom
## Residual deviance: 496.91  on 405  degrees of freedom
## AIC: 502.91
## 
## Number of Fisher Scoring iterations: 4
```

```
exp(coefficients(model_no2f))
```

```
##                          (Intercept)                     sesBelow average 
##                            2.0941176                            1.7844471 
## sesDo not know/do not wish to answer 
##                            0.7731407
```

```
exp(confint(model_no2f))
```

```
##                                         2.5 %   97.5 %
## (Intercept)                          1.623032 2.723355
## sesBelow average                     1.027423 3.219121
## sesDo not know/do not wish to answer 0.426007 1.428396
```

#### interaction term ses\*sex

##### anxiety

```
#model: interaction ses*sex
model_no_interact1 <- glm(hscl_anx_dich ~ ses*sex, family = "binomial", data = data_no)
summary(model_no_interact1)
```

```
## 
## Call:
## glm(formula = hscl_anx_dich ~ ses * sex, family = "binomial", 
##     data = data_no)
## 
## Coefficients:
##                                              Estimate Std. Error z value
## (Intercept)                                   1.13442    0.14370   7.894
## sesBelow average                              1.19286    0.39721   3.003
## sesDo not know/do not wish to answer          0.03830    0.34840   0.110
## sexMale                                      -0.92533    0.25055  -3.693
## sesBelow average:sexMale                      0.41721    0.62737   0.665
## sesDo not know/do not wish to answer:sexMale  0.04029    0.67467   0.060
##                                              Pr(>|z|)    
## (Intercept)                                  2.92e-15 ***
## sesBelow average                             0.002673 ** 
## sesDo not know/do not wish to answer         0.912467    
## sexMale                                      0.000221 ***
## sesBelow average:sexMale                     0.506041    
## sesDo not know/do not wish to answer:sexMale 0.952378    
## ---
## Signif. codes:  0 '***' 0.001 '**' 0.01 '*' 0.05 '.' 0.1 ' ' 1
## 
## (Dispersion parameter for binomial family taken to be 1)
## 
##     Null deviance: 630.39  on 560  degrees of freedom
## Residual deviance: 591.94  on 555  degrees of freedom
## AIC: 603.94
## 
## Number of Fisher Scoring iterations: 4
```

```
exp(coefficients(model_no_interact1))
```

```
##                                  (Intercept) 
##                                    3.1093750 
##                             sesBelow average 
##                                    3.2964819 
##         sesDo not know/do not wish to answer 
##                                    1.0390414 
##                                      sexMale 
##                                    0.3964006 
##                     sesBelow average:sexMale 
##                                    1.5177225 
## sesDo not know/do not wish to answer:sexMale 
##                                    1.0411145
```

```
exp(confint(model_no_interact1))
```

```
##                                                  2.5 %    97.5 %
## (Intercept)                                  2.3609703 4.1511588
## sesBelow average                             1.5956930 7.7241339
## sesDo not know/do not wish to answer         0.5368918 2.1244573
## sexMale                                      0.2422827 0.6481344
## sesBelow average:sexMale                     0.4451527 5.3845663
## sesDo not know/do not wish to answer:sexMale 0.2763860 4.0072723
```

##### depression

```
#model: interaction ses*sex
model_no_interact2 <- glm(hscl_dep_dich ~ ses*sex, family = "binomial", data = data_no)
summary(model_no_interact2)
```

```
## 
## Call:
## glm(formula = hscl_dep_dich ~ ses * sex, family = "binomial", 
##     data = data_no)
## 
## Coefficients:
##                                              Estimate Std. Error z value
## (Intercept)                                    0.7391     0.1318   5.606
## sesBelow average                               0.5791     0.2900   1.997
## sesDo not know/do not wish to answer          -0.2573     0.3073  -0.837
## sexMale                                       -0.7808     0.2430  -3.213
## sesBelow average:sexMale                       0.1908     0.4813   0.396
## sesDo not know/do not wish to answer:sexMale   0.8868     0.6687   1.326
##                                              Pr(>|z|)    
## (Intercept)                                  2.07e-08 ***
## sesBelow average                              0.04583 *  
## sesDo not know/do not wish to answer          0.40239    
## sexMale                                       0.00131 ** 
## sesBelow average:sexMale                      0.69181    
## sesDo not know/do not wish to answer:sexMale  0.18484    
## ---
## Signif. codes:  0 '***' 0.001 '**' 0.01 '*' 0.05 '.' 0.1 ' ' 1
## 
## (Dispersion parameter for binomial family taken to be 1)
## 
##     Null deviance: 722.20  on 560  degrees of freedom
## Residual deviance: 702.47  on 555  degrees of freedom
## AIC: 714.47
## 
## Number of Fisher Scoring iterations: 4
```

```
exp(coefficients(model_no_interact2))
```

```
##                                  (Intercept) 
##                                    2.0941176 
##                             sesBelow average 
##                                    1.7844471 
##         sesDo not know/do not wish to answer 
##                                    0.7731407 
##                                      sexMale 
##                                    0.4580371 
##                     sesBelow average:sexMale 
##                                    1.2102205 
## sesDo not know/do not wish to answer:sexMale 
##                                    2.4272370
```

```
exp(confint(model_no_interact2))
```

```
##                                                  2.5 %    97.5 %
## (Intercept)                                  1.6230317 2.7233546
## sesBelow average                             1.0274228 3.2191214
## sesDo not know/do not wish to answer         0.4260070 1.4283958
## sexMale                                      0.2837776 0.7370214
## sesBelow average:sexMale                     0.4716122 3.1321743
## sesDo not know/do not wish to answer:sexMale 0.6687701 9.5420324
```
