# Supplementary material for: Association of pre-migration socioeconomic status and post-migration mental health in Syrian refugees in Lebanon: a descriptive sex-stratified cross-sectional analysis
Source: Glob Health Res Policy. 2024 Mar 4;9:9. doi: 10.1186/s41256-024-00347-0 (PMC10910804; doi:10.1186/s41256-024-00347-0)
Supplement: Supplementary file 3 — Additional file 3: Supplement 1. Discussion of missingness. Supplement 2. Baseline characteristics of participants included in the main analysis. Supplement 3. Examination of associations between SES and education variables. Supplement 4. Results of sensitivity analyses. [file 41256_2024_347_MOESM3_ESM.pdf]

Additional File 3

Supplement 1: Discussion of Missingness

Missing data patterns were assessed to determine the most likely missingness mechanism for each variable. A distinction was made between missing completely at random (MCAR), i.e., the missing of an observation being independent of everything, that is, a truly random process that led to missingness; missing at random (MAR), i.e., missing of an observation being conditionally independent, that is, independent of the missing variable but dependent on other observed variables; and missing not at random (MNAR), i.e., missing of an observation depending on the missing value itself or other unknown reasons (Donders et al., 2006).

For the HSCL-25 score variables, missing in both HSCL-25 sub-scores occurred in 45 cases, with 54 missing data points in total for the HSCL-25 anxiety score and 120 for the HSCL-25 depression score. This indicates data being MAR, or MNAR if missingness also depends on unobserved variables. To discuss the plausibility of MNAR, it is necessary to combine exploratory techniques with subject-matter knowledge and knowledge of the data collection process. Therefore, in addition, missing data across variables were also visualized (see Figures A1 and A2).

As missing of observations on SES might be informative and hence not ignorable, missing observations have been neither left out nor imputed, but were included as a separate category. Regarding missing observations in the HSCL-25 sub-scores, MNAR would mean that non-response in questions on mental health depended on the mental health status itself. Several studies examining the correlation between (mental) health status and nonresponse suggest that nonresponse is often correlated to worse mental health, but differences due to the mental health status alone are fairly small and additionally due to other factors (Torvik et al., 2012; Vink et al., 2004). While this speaks against MNAR in these variables, it cannot be entirely ascertained whether observations are MAR or MNAR.

Therefore, in a sensitivity analysis that tested the case of violation of the MAR assumption in the anxiety and depression score, possible extreme cases were explored by forcing values into either of the binary categories on the HSCL-25 score. In another sensitivity analysis, different thresholds for the HSCL-25 sub-scores that were ascertained in an Arab study population were used. Both sensitivity analyses yielded results that were similar to the main analysis, supporting the robustness of the general results of this study.

Figure A1: Distribution of missing data points across SES levels.

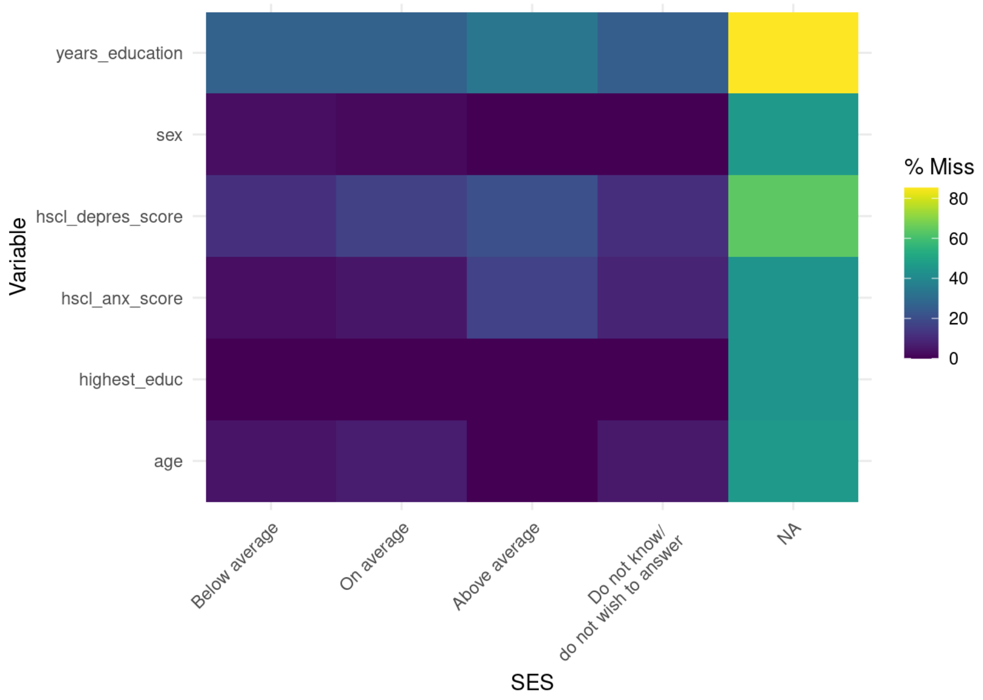

Note: This figure was created using the *gg_miss_fct* function of the *naniar* package in R. The colors denote the percentage of missing data points per variable (on the vertical axis), stratified by SES (on the horizontal axis).

Figure A2: Connections of missing data points across variables.

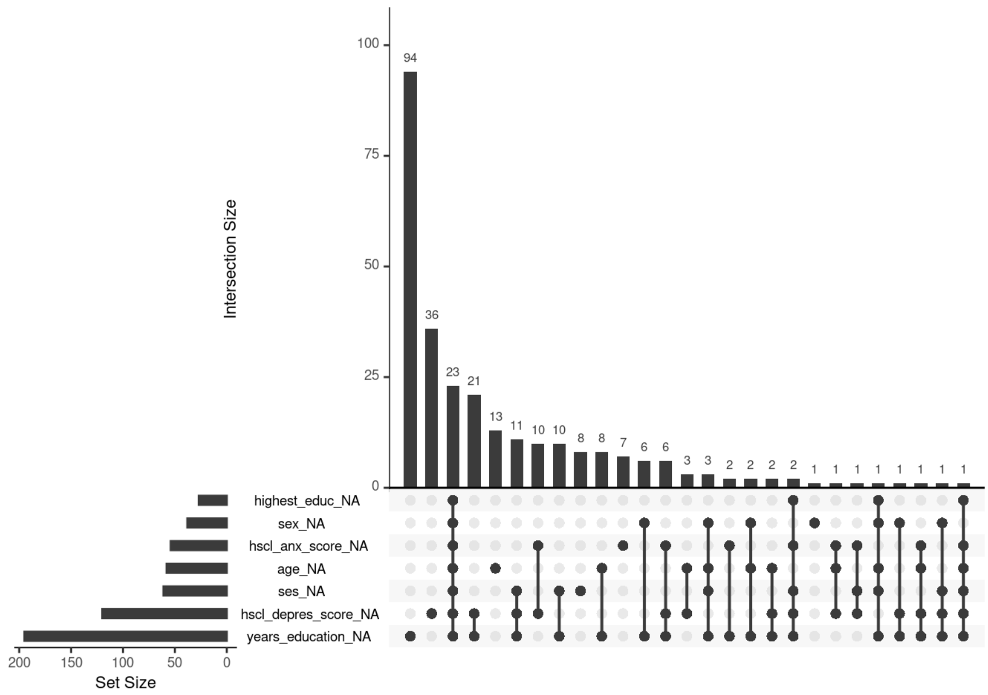

Note: This figure was created using the *gg_miss_upset* function of the *naniar* package in R. The bars on the left present the number of missing values per listed variable ("Set Size"). The dots and their connections on the right denote (column-wise) missingness patterns of the variables within individuals. The bars above ("Intersection Size") show in how many individuals the specific missingness patterns occurred.

Supplement 2: Baseline Characteristics of Participants Included in the Main Analysis

Variable	Total (n = 457)	*Female* (n = 322)	*Male* (n = 135)
**Age:**			
median [min, max]	35 [18, 72]	34 [18, 71]	38 [18, 72]
Missing: n (%)	21 (4.6%)	20 (6.2%)	1 (0.7%)
**SES:**			
n (%)			
- Below average	118 (25.8%)	78 (24.2%)	40 (29.6%)
- On average	272 (59.5%)	194 (60.2%)	78 (57.8%)
- Above average	18 (3.9%)	13 (4.0%)	5 (3.7%)
- Do not know / do not wish to answer	49 (10.7%)	37 (11.5%)	12 (8.9%)
**Education - years:**			
median [min, max]	6 [0, 21]	6 [0, 20]	6 [0, 21]
Missing: n (%)	112 (24.5%)	81 (25.2%)	31 (23.0%)
**Education - highest degree:**			
n (%)			
- No education	141 (30.9%)	101 (31.4%)	40 (29.6%)
- Primary school	204 (44.6%)	138 (42.9%)	66 (48.9%)
- High school	87 (19.0%)	67 (20.8%)	20 (14.8%)
- Higher	25 (5.5%)	16 (5.0%)	9 (6.7%)
**HSCL-25 anxiety score:**			
median [min, max]	2.40 [1.00, 4.00]	2.50 [1.00, 4.00]	2.10 [1.00, 3.80]
n (%)			
- < 1.75	91 (19.9%)	47 (14.6%)	44 (32.6%)
- ≥1.75	366 (80.1%)	275 (85.4%)	91 (67.4%)
**HSCL-25 depression score:**			
median [min, max]	2.33 [1.00, 4.00]	2.40 [1.00, 4.00]	2.07 [1.00, 3.67]
n (%)			
- < 1.75	98 (21.4%)	48 (14.9%)	50 (37.0%)
- ≥1.75	359 (78.6%)	274 (85.1%)	85 (63.0%)
**Duration of stay in Lebanon (months):**			
median [min, max]	8.0 [2.0, 12.0]	8.0 [2.0, 12.0]	8.0 [4.0, 12.0]

Supplement 3: Examination of Associations between SES and Education Variables

Figure A3: Boxplot for years of education by SES category with overlaid observations.

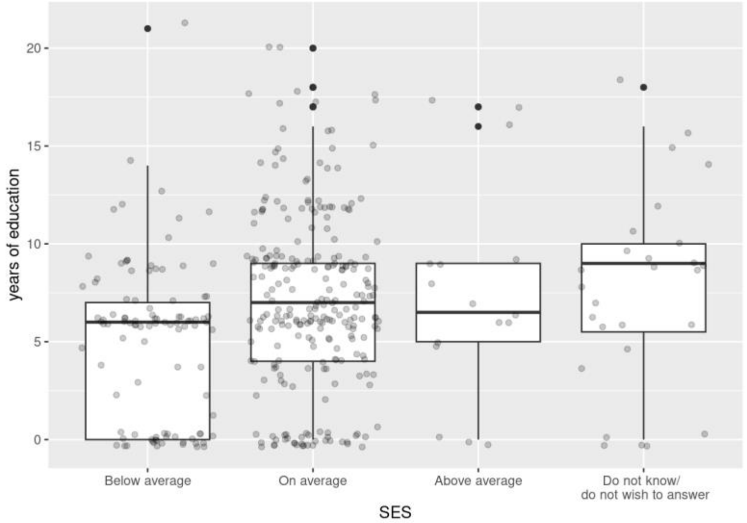

Figure A4: Distribution of highest education level by SES category.

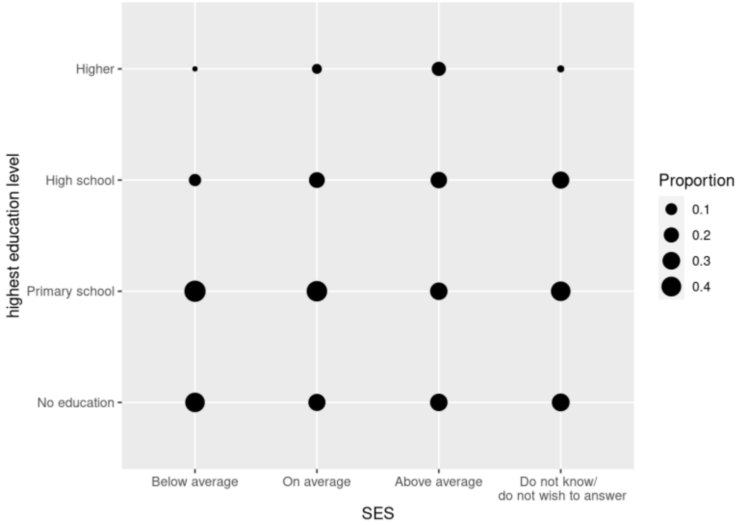

Figure A5: Boxplot for years of education by highest education level achieved with overlaid observations.

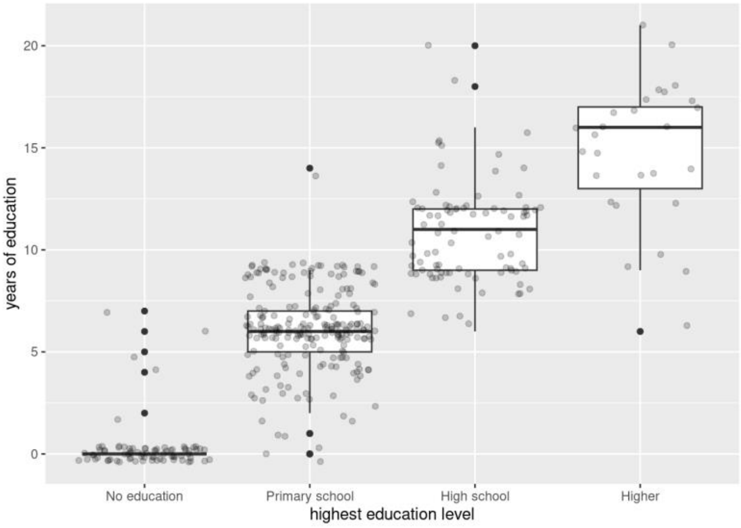

Supplement 4: Results of Sensitivity Analyses

Table A1: Sensitivity analysis with different threshold: ORs for HSCL-25 anxiety score ≥ 2.0 by SES, total and stratified by sex.

		**Total**		**Female**		**Male**	
		OR [95% CI]	*p*-value	OR [95% CI]	*p*-value	OR [95% CI]	*p*-value
**SES**	Below average	1.75 [1.06, 2.96]	0.032	1.60 [0.83, 3.26]	0.176	2.46 [1.11, 5.66]	0.030
	On average	1 (ref)	-	1 (ref)	-	1 (ref)	-
	Above average	1.65 [0.57, 5.94]	0.392	1.07 [0.31, 4.90]	0.925	4.21 [0.59, 84.37]	0.208
	Do not know / do not wish to answer	1.83 [0.91, 4.04]	0.108	2.64 [0.98, 9.18]	0.081	1.05 [0.30, 3.64]	0.934

Table A2: Sensitivity analysis with different threshold: ORs for HSCL-25 depression score ≥ 2.1 by SES, total and stratified by sex.

		**Total**		**Female**		**Male**	
		OR [95% CI]	*p*-value	OR [95% CI]	*p*-value	OR [95% CI]	*p*-value
**SES**	Below average	2.06 [1.29, 3.34]	0.003	2.52 [1.35, 4.98]	0.005	1.94 [0.90, 4.26]	0.091
	On average	1 (ref)	-	1 (ref)	-	1 (ref)	-
	Above average	1.15 [0.44, 3.21]	0.778	0.88 [0.28, 3.02]	0.833	2.16 [0.34, 17.08]	0.414
	Do not know / do not wish to answer	1.26 [0.68, 2.40]	0.469	1.15 [0.55, 2.50]	0.714	1.44 [0.42, 4.99]	0.559

Table A3: Sensitivity analysis: ORs for experiencing anxiety symptoms (HSCL-25 ≥ 1.75) depending on SES, total and stratified by sex (extreme scenario 2: all missings in HSCL-25 score set to < 1.75)

		**Total**		**Female**		**Male**	
		OR [95%CI]	*p*-value	OR [95%CI]	*p*-value	OR [95%CI]	*p*-value
**SES**	Below average	3.51 [1.98, 6.67]	<0.001	3.11 [1.49, 7.31]	0.005	5.16 [2.10, 14.68]	<0.001
	On average	1 (ref)	-	1 (ref)	-	1 (ref)	-
	Above average	0.69 [0.30, 1.69]	0.396	0.48 [0.18, 1.35]	0.143	1.67 [0.31, 12.51]	0.564
	Do not know / do not wish to answer	1.09 [0.62, 1.98]	0.775	0.98 [0.50, 2.01]	0.953	1.12 [0.36, 3.64]	0.850

Table A4: Sensitivity analysis: ORs for experiencing depressive symptoms (HSCL-25 ≥ 1.75) depending on SES, total and stratified by sex (extreme scenario 2: all missings in HSCL-25 score set to < 1.75)

		**Total**		**Female**		**Male**	
		OR [95%CI]	*p*-value	OR [95%CI]	*p*-value	OR [95%CI]	*p*-value
**SES**	Below average	1.78 [1.14, 2.83]	0.012	1.75 [1.00, 3.16]	0.057	2.17 [1.03, 4.73]	0.046
	On average	1 (ref)	-	1 (ref)	-	1 (ref)	-
	Above average	0.82 [0.36, 1.96]	0.649	0.73 [0.28, 2.06]	0.538	1.05 [0.18, 5.91]	0.958
	Do not know / do not wish to answer	0.97 [0.57, 1.68]	0.917	0.76 [0.41, 1.40]	0.367	1.88 [0.60, 6.53]	0.289

Table A5: Sensitivity analysis: ORs for experiencing anxiety symptoms (HSCL-25 ≥ 1.75) depending on SES, total and stratified by sex (extreme scenario 1: all missings in HSCL-25 score set to ≥ 1.75)

		**Total**		**Female**		**Male**	
		OR [95%CI]	*p*-value	OR [95%CI]	*p*-value	OR [95%CI]	*p*-value
**SES**	Below average	3.99 [2.09, 8.43]	<0.001	2.52 [1.16, 6.34]	0.031	10.20 [3.38, 44.31]	<0.001
	On average	1 (ref)	-	1 (ref)	-	1 (ref)	-
	Above average	1.23 [0.48, 3.81]	0.688	0.75 [0.25, 2.72]	0.619	3.82 [0.59, 74.84]	0.229
	Do not know / do not wish to answer	1.54 [0.81, 3.14]	0.207	1.74 [0.75, 4.76]	0.233	1.02 [0.33, 3.32]	0.973

Table A6: Sensitivity analysis: ORs for experiencing depressive symptoms (HSCL-25 ≥ 1.75) depending on SES, total and stratified by sex (extreme scenario 1: all missings in HSCL-25 score set to ≥ 1.75)

		**Total**		**Female**		**Male**	
		OR [95%CI]	*p*-value	OR [95%CI]	*p*-value	OR [95%CI]	*p*-value
**SES**	Below average	1.63 [0.94, 2.93]	0.091	1.91 [0.86, 4.85]	0.136	1.77 [0.80, 4.09]	0.167
	On average	1 (ref)	-	1 (ref)	-	1 (ref)	-
	Above average	0.97 [0.37, 3.00]	0.950	0.81 [0.25, 3.61]	0.743	1.21 [0.22, 9.09]	0.828
	Do not know / do not wish to answer	2.26 [1.05, 5.61]	0.053	2.05 [0.77, 7.12]	0.191	2.23 [0.64, 10.36]	0.244
